# Modified gefitinib conjugated Fe_3_O_4_ NPs for improved delivery of chemo drugs following an image-guided mechanistic study of inner vs. outer tumor uptake for the treatment of non-small cell lung cancer

**DOI:** 10.3389/fbioe.2023.1272492

**Published:** 2023-10-09

**Authors:** Suresh Thangudu, Ching-Yi Tsai, Wei-Che Lin, Chia-Hao Su

**Affiliations:** ^1^ Center for General Education, Chang Gung University, Taoyuan, Taiwan; ^2^ Canary Center for Cancer Early Detection, Department of Radiology, Stanford University School of Medicine, Palo Alto, CA, United States; ^3^ Institute for Translational Research in Biomedicine, Kaohsiung Chang Gung Memorial Hospital, Kaohsiung, Taiwan; ^4^ Department of Diagnostic Radiology, Kaohsiung Chang Gung Memorial Hospital, Kaohsiung, Taiwan; ^5^ Department of Biomedical Imaging and Radiological Sciences, National Yang-Ming Chiao Tung University, Taipei, Taiwan; ^6^ Department of Radiation Oncology, Kaohsiung Chang Gung Memorial Hospital, Kaohsiung, Taiwan

**Keywords:** iron oxide NPs, modified gefitinib, non-small cell lung cancer, drug delivery, magnetic resonance imaging

## Abstract

Gefitinib (GEF) is an FDA-approved anti-cancer drug for the first-line treatment of patients with metastatic non-small cell lung cancer (NSCLC). However, the efficacy of anticancer drugs is limited due to their non-specificity, lower accumulation at target sites, and systemic toxicity. Herein, we successfully synthesized a modified GEF (mGEF) drug and conjugated to Iron oxide nanoparticles (Fe_3_O_4_ NPs) for the treatment of NSCLC via magnetic resonance (MR) image-guided drug delivery. A traditional EDC coupling pathway uses mGEF to directly conjugate to Fe_3_O_4_ NPs to overcom the drug leakage issues. As a result, we found *in vitro* drug delivery on mGEF- Fe_3_O_4_ NPs exhibits excellent anticancer effects towards the PC9 cells selectively, with an estimated IC 50 value of 2.0 μM. Additionally, *in vivo* MRI and PET results demonstrate that the NPs could accumulate in tumor-specific regions with localized cell growth inhibition. Results also revealed that outer tumor region exhibiting a stronger contrast than the tinner tumor region which may due necrosis in inner tumor region. *In vivo* biodistribution further confirms Fe_3_O_4_ NPs are more biocompatible and are excreated after the treatment. Overall, we believe that this current strategy of drug modification combined with chemical conjugation on magnetic NPs will lead to improved cancer chemotherapy as well as understanding the tumor microenvironments for better therapeutic outcomes.

## 1 Introduction

**SCHEME 1:**
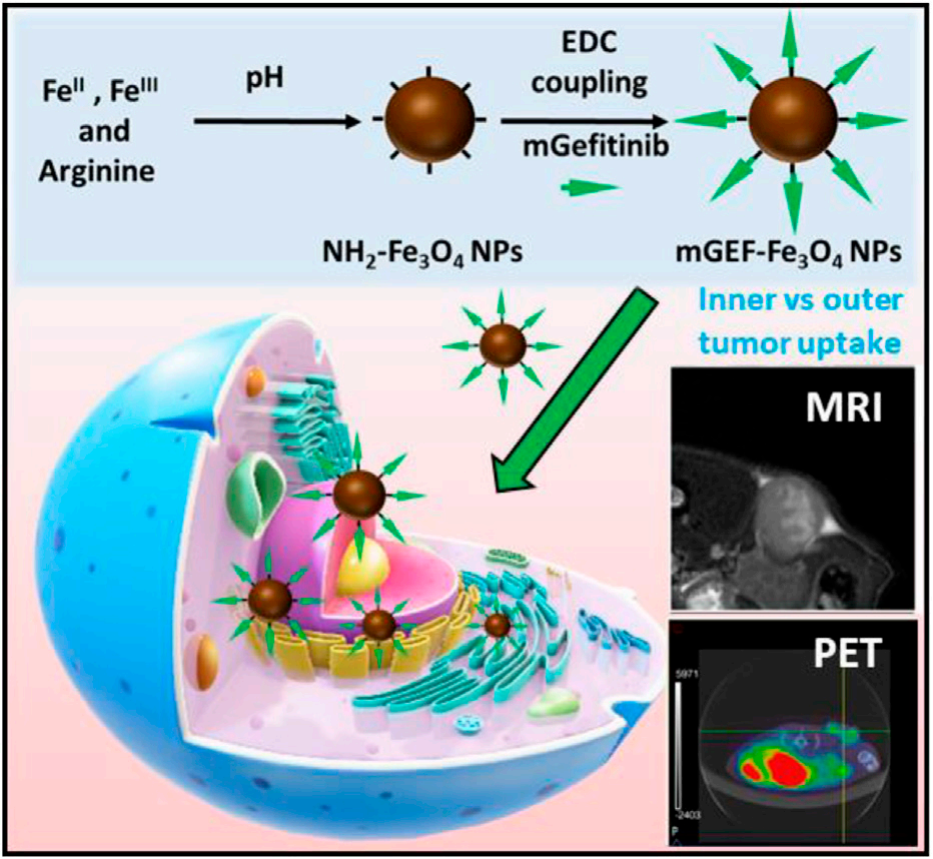
Schematic illustration of chemically conjugated modified gefitinib drug to Fe_3_O_4_ NPs for image-guided chemo delivery for NSCLC treatment.

Lung carcinoma is one of the major causes of cancer death in both men and women worldwide (Pakzad et al., 2015; Sung et al., 2021). Specifically, NSCLC grows rapidly and metastasizes easily through the lymph nodes to the whole body, especially to the brain, liver, and bone (D'Antonio et al., 2014; Ren et al., 2016; Ko et al., 2021). Therefore, surgery is not a suitable treatment option for NSCLC except for early solid tumors. Subsequently, combined therapeutic approaches such as surgical removal of the tumor followed by cytotoxic chemotherapy and radiation therapy were employed (Miller et al., 2022). However, chemo and radiation therapy affect normal tissue along with tumorous tissues. To further improve the efficacy of chemotherapeutic treatment and reduce toxicity, targeting therapy selectively targets the tumor site and mediates the therapeutic effects (Baudino, 2015; Zhong et al., 2021). Thus, selective targeting of chemo drugs to the tumor site is an emerging choice in clinical applications. Most of the targeted therapy mechanism involves targeting the epidermal growth factor receptor (EGFR) family inhibitors, angiogenesis inhibitors, signal transduction inhibitors, apoptosis inducers, eicosanoid pathway inhibitors, etc (Chan and Hughes, 2015; Mustachio and Roszik, 2020). EGFR-targeted therapy is emerging as a hot topic in clinical cancer treatment, especially for treating NSCLC (Antonicelli et al., 2013; Yuan et al., 2019; Li et al., 2023). Patient meta-analysis found EGFR-targeting TKI to be effective in nearly 70% of patients with EGFR mutations, but effective in only 10% of patients with wild-type EGFR (Mitsudomi et al., 2006). The FDA approved the GEF drug (Iressa, Astra Zeneca Pharmaceuticals) which enhances the apoptosis of tumor cells and then inhibits tumor growth (Dyer, 2003). Specifically, GEF is a tyrosine kinase inhibitor that targets the EGFR and induces dramatic clinical responses in NSCLCs with activating mutations within the EGFR kinase domain (Sordella et al., 2004; Kazandjian et al., 2016). However, frequent adverse effects related to GEF drug include diarrhea, acne-like rash, gastrointestinal effects, dry-skin, nausea and vomiting, etc. (Cersosimo, 2006). Besides, GEF applications are further limited by its low water solubility, stability, and utilization rate (Godugu et al., 2016; Liu et al., 2018). Therefore, it is highly desirable to develop efficient drug carriers for the successful delivery of GEF to lung cancer cells specifically. Inorganic and polymeric nanocarrier-mediated drug delivery systems have received greater attention to address these aforementioned issues (Shi et al., 2020; Edis et al., 2021; Gagliardi et al., 2021). Active drug compounds encapsulated in nanocarriers offer excellent bioavailability, prevent degradation, reduce toxic effects, control drug release, and target drug delivery. Most drug loading strategies were either physical loading/incorporation or electrostatic interactions (Shanmugam et al., 2014; Su and Cheng, 2015; Su et al., 2016; Tsai et al., 2020). For instance, Lee et al. developed a GEF–cyclodextrin complex and Trummer et al. developed a nano liposomal GEF formulation to further improve the solubility rate of GEF drugs (Phillip Lee et al., 2009; Trummer et al., 2012). Subsequently, for the selective targeting of the tumor site, Shi et al. fabricated GEF-loaded folate-decorated bovine serum albumin-conjugated carboxymethyl-b-cyclodextrin NPs (FA-BSA-CM-β-CD NPs) which offer significantly enhanced drug delivery and attenuated autophagy in folate receptor-positive cancer cells (Shi et al., 2014). A nanographene oxide (NGO) based drug delivery of GEF approach was later developed for the treatment of lung cancer, promoting the accumulation of nanosheets in tumor sites and facilitating drug release from the nanosheets in response to tumor-relevant GSH (Liu et al., 2018). Later on, several nanocarrier studies sought to deliver a GEF drug to tumor sites, including Gefitinib-bound Carbon dots (DFO-Gef-C′ dots) (Madajewski et al., 2020), Gefitinib encapsulated apoferritin (Kuruppu et al., 2015), Gefitinib-coated AuNPs (Lam et al., 2014), and GEF loaded into p28-functionalized PLGA NPs (Garizo et al., 2021). Recently, to further improve the blood circulation time and targeting ability, Wen et al., fabricated a biomimetic R-RBC@GEF-NPs nano-system by encapsulating GEF-loaded albumin NPs into cRGD-modified red blood cell (RBC) membranes for treating lung cancer (Wen et al., 2021). However, insufficient drug loading and uncontrolled release of drugs from nanocarriers hinder successful translation into clinical applications (Liu et al., 2020). Most drug delivery-based nanocarriers (liposomes, porous materials, etc.) cannot be used because the drugs leak during blood circulation before reaching their target sites, resulting in lower accumulation of drugs at tumor sites and lower therapeutic efficacy (Chen et al., 2020; Feng et al., 2023). Besides, monitoring the therapeutic prognosis or therapeutic delivery of drugs is highly desirable to further promote therapeutic efficacy (Solorio et al., 2010; Haris et al., 2015; Ojha et al., 2015; Gao et al., 2018; Thangudu et al., 2021). This raises the need for efficient image-guided nanocarrier systems for the simultaneous monitoring of the therapeutic site and sustained delivery of chemo drugs for treating NSCLC. To this end, the present study describes the successful synthesis of a modified GEF drug which is chemically conjugated with the Fe_3_O_4_ NPs (mGEF@ Fe_3_O_4_ NPs) for simultaneous MR image-guided targeted chemotherapy for lung cancer treatment. Direct chemical conjugation of mGEF on biocompatible IONPs, significantly reducing drug leakage problems and enhancing the uptake of chemo drugs by tumor cells. *In vitro* results indicate successful delivery of mGEF on IONPs into the tumor cells via endocytosis pathway resulting in improved cancer cell death. *In vivo,* MR imaging studies reveal that drug uptake to the outer region of the tumor is greater than that to the inner region due to tumor necrosis in the inner region. Furthermore, results of *in vivo* biodistribution assays reveal the present IONPs possess excellent biocompatibility with no noticeable side effects on major organs. To the best of our knowledge, the delivery of chemically conjugated drugs on nanocarriers against NSCLCs has been seldom reported. A detailed illustration of the present work is shown in Scheme 1.

## 2 Results and discussion

### 2.1 Preparation and characterization of GEF-modified Fe_3_O_4_ NPs (mGEF@Fe_3_O_4_ NPs)

**FIGURE 1:**
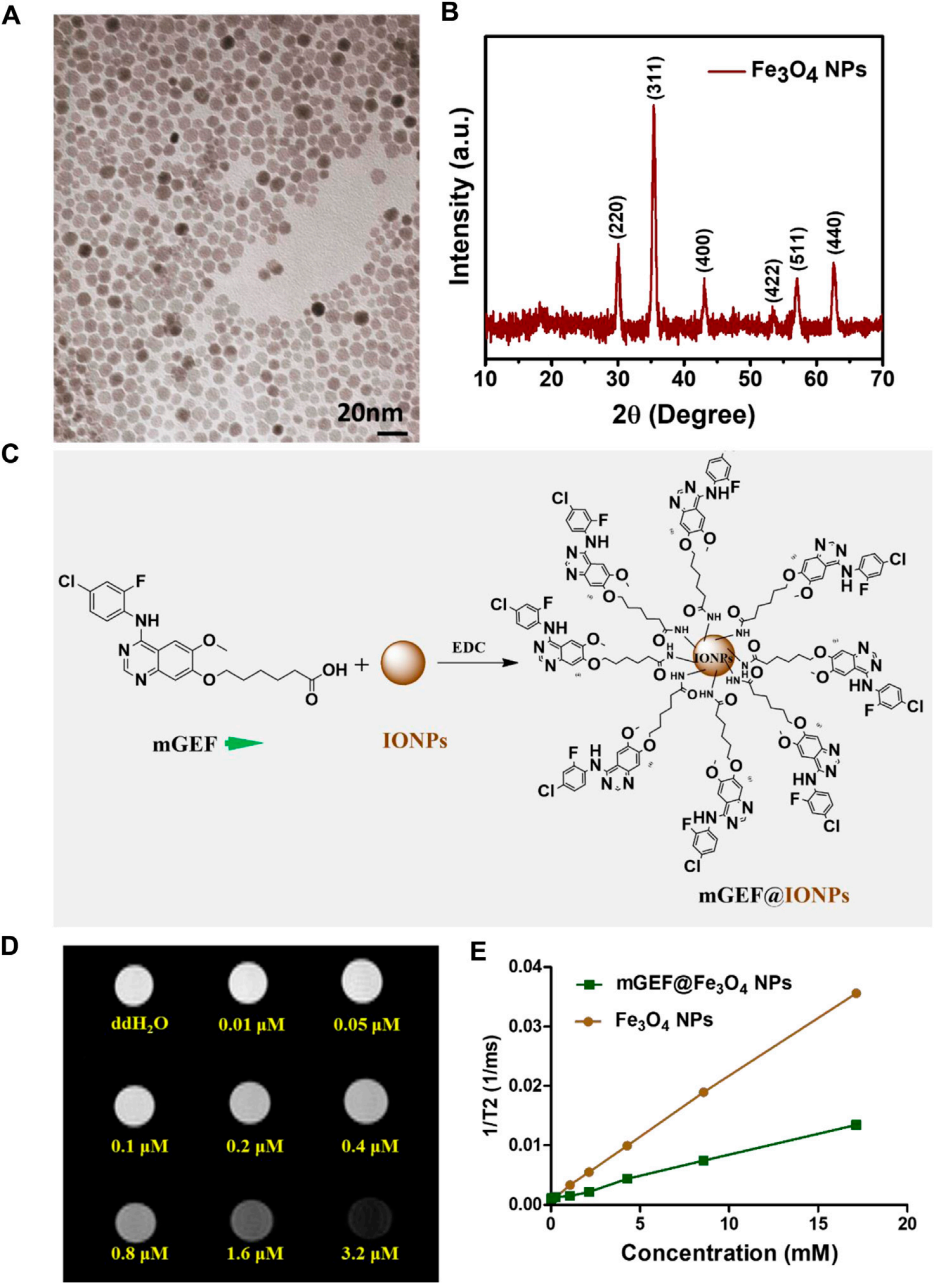
Synthesis and characterization of Fe_3_O_4_ NPs. **(A)** HR-TEM image of Fe_3_O_4_ NPs. **(B)** XRD spectra of Fe_3_O_4_ NPs. **(C)** Schematic representation of surface conjugated Fe_3_O_4_ NPs with mGEF. **(D)** Concentration dependent T2 weighed MRI phantom images of Fe_3_O_4_ NPs. **(E)** 1/T2 kinetic plot of bare Fe_3_O_4_ NPs and mGEF@ Fe_3_O_4_ NPs.

NH_2_-terminated Fe_3_O_4_ NPs were synthesized using a previously reported protocol (Su et al., 2016). The as-synthesized NPs exhibit a clear morphology and uniform size distribution, confirmed by high-resolution transmission electron microscopy (HR-TEM) analysis, as shown in Figure 1A. The average size of the NPs is estimated to be around 6 nm. The crystallinity and phase formation of NPs was confirmed by XRD analysis, explicitly demonstrating the successful formation of Fe_3_O_4_ NPs. Figure 1B shows peaks at 30.1°, 35.5°, 43.1°, 53.5°, 57.4°, and 62.5°, which are respectively indexed to reflections from the (220), (311), (400), (422), (511), and (440) faces of the inverse spinel iron oxide (JCPDS No. 19-0629). The NH_2_ functional group on the NP surface result in their hydrophilic nature and facilitates an even dispersion in an aqueous medium. The -NH_2_ functional group on the NPs also facilitates further conjugation of targeting ligands on the NP’s surface. On the other hand, non-specific and insufficient delivery of GEF to the therapeutic site is a critical issue in the treatment of lung cancer. To address this issue, we first synthesized the GEF drug via a three-step process, and the obtained products were confirmed by ^1^H NMR analysis (the synthesis process is shown in supporting information, steps 1-3). Before the conjugation of GEF on IONPs, we modified the GEF structure with the terminal carboxyl group by introducing/substituting the 6-propylmorpholino group on one side of the carbon chain of GEF (see step 4 in supporting information) which is the weakest binding with kinase. The other side of GEF remains the same with the presence of active binding site 1-N group on the quinazoline ring. Successful modification of GEF was confirmed by ^1^H-NMR analysis (Supplementary Figure S1). Finally, -NH_2_ functionalized Fe_3_O_4_ NPs were successfully conjugated with the terminal group of–COOH on modified the GEF (mGEF) drug via 1-Ethyl-3-(3-dimethyl aminopropyl)carbodiimide (EDC) coupling. The schematic representation of the conjugation procedure is shown in Figure 1C. Successful formation of mGEF conjugated Fe_3_O_4_ NPs (mGEF@Fe_3_O_4_ NPs) was confirmed by UV-visible absorption spectra analysis. Absorption spectra of mGEF are specifically located at 340 nm wavelength whereas in bare NPs there is no significant absorption at 340 nm. In contrast, after modifying the Fe_3_O_4_ NPs with mGEF (mGEF@ Fe_3_O_4_ NPs), we observed significant absorption at 340 nm wavelength which strongly confirms the successful conjugation of mGEF on NPs (Supplementary Figure S2). Subsequently, different concentrations (low to high) of mGEF were added to Fe_3_O_4_ NPs and we monitored the changes in absorbance. Results reveal the significant enhancement of absorption intensity at 340 nm wavelength by increasing the mGEF concentration on NPs (Supplementary Figure S3). In addition, Iron-based NPs are proven T2-weighed MRI contrast agents and display excellent MR contrast abilities both *in vitro* and *in vivo* (Shen et al., 2017; Thangudu et al., 2022b; Thangudu et al., 2023). To verify the contrast enhancement efficiency of present synthesized-Fe_3_O_4_ NPs, T2-weighted MRI analysis was performed with a 7.0 T MRI scanner. The samples were serial diluted with various concentrations of Fe_3_O_4_ NPs and suspended in deionized water. Both the gradient-echo-based and spin-echo-based T2-weighted imaging showed significant enhancement in a concentration-dependent manner. As the concentration of NPs increased, the samples grew increasingly dark, corresponding to the enhanced T2 contrast compared to the deionized water alone. We also verified the MR activities of mGEF@Fe_3_O_4_ NPs, and noticed that the MR contrast abilities of mGEF-Fe_3_O_4_ NPs slightly less than bare Fe_3_O_4_ NPs alone, which may be attributed due to the conjugation of mGEF on the NPs surface possibly covering the NPs surface and altering the magnetic field-water interaction, resulting in a slight decrease in the paramagnetic effect (Liang et al., 2016).

### 2.2 *In vitro* targeting and chemotherapeutic efficiency of mGEF@Fe_3_O_4_ NPs

**FIGURE 2:**
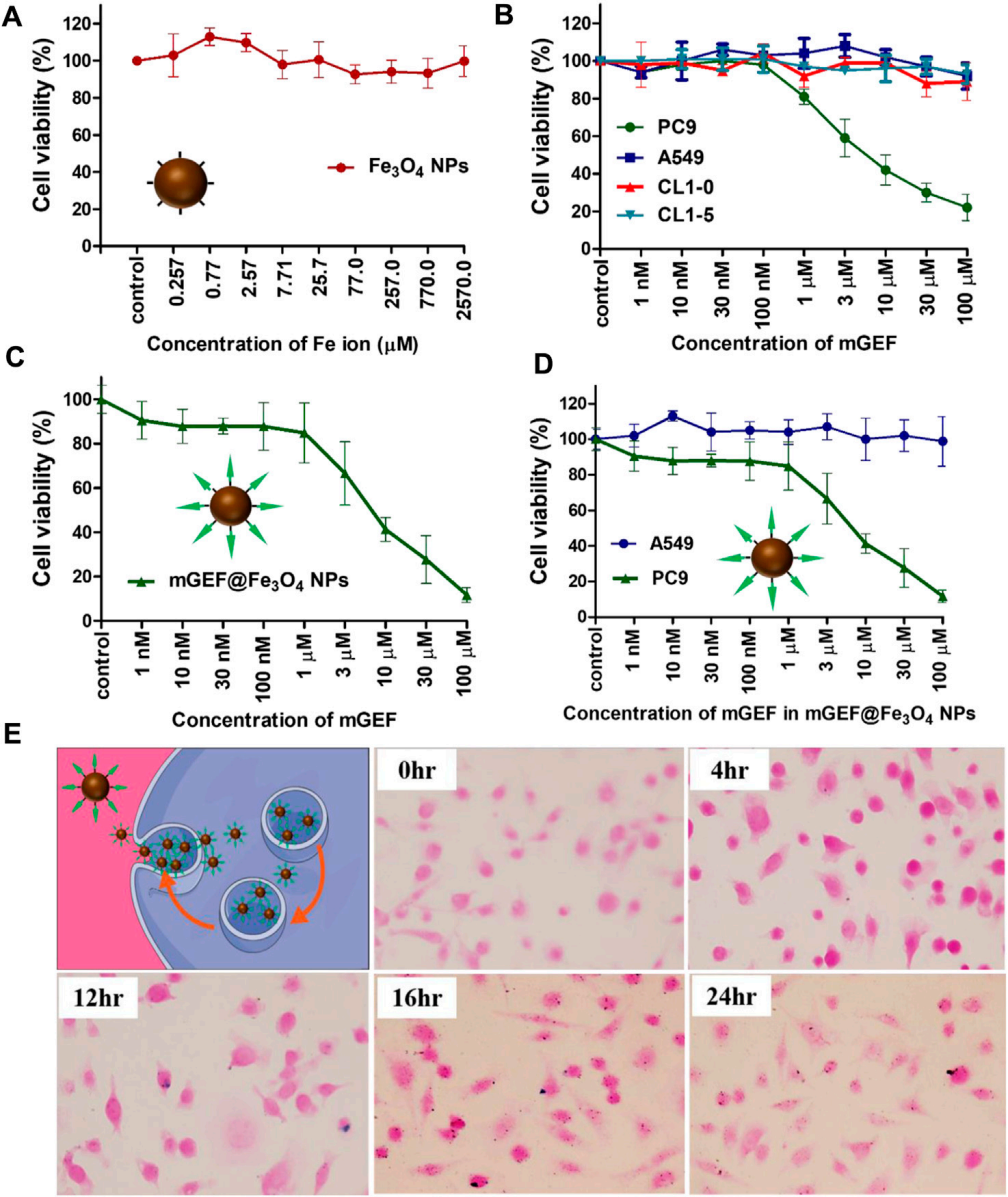
*In vitro* drug efficiency studies on mGEF@Fe_3_O_4_ NPs. **(A)** *In vitro* cytotoxicity assay of bare Fe_3_O_4_ NPs at different concentrations in PC9 cells. **(B)** *In vitro* drug efficiency and targeting ability of mGEF on various cell lines. **(C)** *In vitro* drug efficiency of mGEF and mGEF@Fe_3_O_4_ NPs. **(D)** Drug efficiency study of mGEF@Fe_3_O_4_ NPs to PC9 (L858R mutant EGFR) and A549 (wild-type EGFR) cells. **(E)** Perls’ blue staining assay to confirm the endocytosis of NPs. Three repetitions of the experiment are used to calculate the standard deviation taht represent the error bars on the each graphs.

Following the effective synthesis of mGEF@Fe_3_O_4_ NPs, *in vitro* studies were carried out to assess the therapeutic efficacy of mGEF@Fe_3_O_4_ NPs for treating non-small cell lung cancer. The cytotoxicity of NPs was investigated prior to the *in vitro* therapeutic evaluation. Results of 3-(4,5-dimethylthiazol-2-yl)-2,5-diphenyltetrazolium bromide (MTT) cytotoxicity experiments show that cancer cells’ *in vitro* viability is unaffected by exposure to Fe_3_O_4_ NPs at concentrations ranging from 0.257 μM to 2570 μM (Figure 2A). Cytotoxicity results indicate that the as-synthesized Fe_3_O_4_ NPs are biocompatible. Previous work also notes that Fe_3_O_4_ NPs are biocompatible and have been investigated in several biomedical applications (Martins et al., 2021; Montiel Schneider et al., 2022). Notably, GEF is a tyrosine kinase inhibitor that has been given clinical approval to treat NSCLC (Kitazaki et al., 2005; Kazandjian et al., 2016). As a proof of concept, we conducted *in vitro* cell survival tests utilizing mGEF@Fe_3_O_4_ NPs to further demonstrate the targeting capability of GEF. To determine the targeting capabilities of mGEF, we used two distinct cell lines. PC9 is one of the NSCLC cell lines most vulnerable to GEF, while EGFR wild-type cell lines like A549, CL1-10, and CL1-5 are resistant to GEF. As a result, modified GEF successfully inhibited the PC9 cell survival and IC 50 was estimated to be around 5 μM, indicating the good targeting ability of mGEF (Figure 2B). In other cell lines, cell viability remains unchanged under the same circumstances due to GEF resistantance. Due to the fact that the modified drug was dissolved in DMSO for cell viability tests, we also examined the same concentration of DMSO by itself to determine whether or not it had any negative effects on the cells. The results showed that the concentration of DMSO used had no harmful effects on cells and caused no changes to cell viability (Supplementary Figure S4). The drug efficiency of mGEF@Fe_3_O_4_ NPs was then verified in PC9 cells via MTT assay. As shown in Figure 2C, cell viability of PC9 cells drastically decreased after treatment with mGEF@Fe_3_O_4_ NPs and the estimated IC 50 value is 2.0 μM. Further we have evaluted the IC50 value of mGEF@Fe3O4 NPs and mGEF drug (Supplementary Figure S5). Precisely, the inhibition efficiency of PC9 cells on mGEF@Fe_3_O_4_ NPs and modified GEF drug alone as follows 2 μM and 500 nM respectively, indicating that drug on NPs shown good therapeutic response similar like mGEF alone due to the rapid release of drug on NPs. We then evaluated the therapeutic effectiveness of Fe3O4@mGEF on PC9 and A549 cancer cells. As anticipated, increased mortality of PC9 cells was seen on mGEF@Fe3O4 NPs under the same experimental circumstances. As a result of A549s resistance to the mGEF@Fe3O4 NPs even at higher concentrations (100 μM), there was no significant cell inhibition (Figure 2D). GEF primarily blocks the tyrosine kinase domain and suppresses signal transmission. These tyrosine kinases will, however, be found inside of cells. Therefore for effective cell inhibition, the nano platform must infiltrate the intracellular setting and deliver the drugs. Consequently, we used Perls’ blue staining assay to determine whether or not the mGEF@Fe_3_O_4_ NPs were successfully endocytosed by the cells. As shown in Figure 2E; Supplementary Figure S6, we observed the presence of Fe_3_O_4_ NPs inside the PC9 cells after treatment with mGEF@Fe_3_O_4_ NPs. mGEF@Fe_3_O_4_ NPs began to transport into the intracellular domain after 4-h incubation and started to accumulate more NPs inside the cells over the prolonged incubation time. The results demonstrate that the current mGEF@Fe_3_O_4_ NPs were successfully endocytosed into the cell and were primarily distributed around the cell membrane, making it possible to block the tyrosine kinase. Mechanistic experiments were then carried out to further understand how exposure to mGEF@Fe_3_O_4_ NPs caused PC9 cells to perish. It is widely known that the primary cause of GEF cell death in EGFR L858R mutant non-small lung cancer is apoptosis (Tracy et al., 2004). In early apoptotic cells, membrane phospholipid phosphatidylserine (PS) is translocated from the inner to the outer leaflet of the plasma membrane, thereby exposing PS to the external cellular environment (Mariño and Kroemer, 2013). Annexin V is a Ca^2+^-dependent phospholipid-binding protein that has a high affinity to bind with exposed PS (Vermes et al., 1995). In late apoptosis, the cell membrane was damaged and became permeable, allowing the nucleic acid dye (Propidium iodide, PI) to be intercalated in nucleic acid. Thus, to distinguish the mechanism of cell death induced by mGEF@Fe_3_O_4_ NPs, we performed Annexin V-FITC and PI fluorescence staining analysis on gefitinib-sensitive PC9 cells as well as gefitinib resistant A549 cells. As shown in Supplementary Figure S7, the cell density of PC9 showed no significant difference from the control group up to treatment with concentrations of 300 nM of mGEF@Fe_3_O_4_ NPs. In contrast, increasing the concentration of mGEF@Fe_3_O_4_ NPs to 100 μM resulted in increased PC9 cell death. On the contrary, we observed no significant response to mGEF@Fe_3_O_4_ NPs for A549 cells, even with dosages of up to 100 μM (Supplementary Figure S8). Although the morphology of the PC9 cell looks undamaged after treatment with 300 nM concentration of NPs, it processed in early apoptosis, as shown in the Annexin V-FITC image. For both Annexin V-FITC or PI staining, A549 cells were still active with reduced fluorescence which respectively represented apoptosis and necrosis. Results of fluorescence microscopy studies verified the mGEF@Fe_3_O_4_ NPs lead cell apoptosis and that it is specific to PC9 cells.

### 2.3 *In vitro* and *in vivo* imaging of mGEF@Fe_3_O_4_ NPs

**FIGURE 3:**
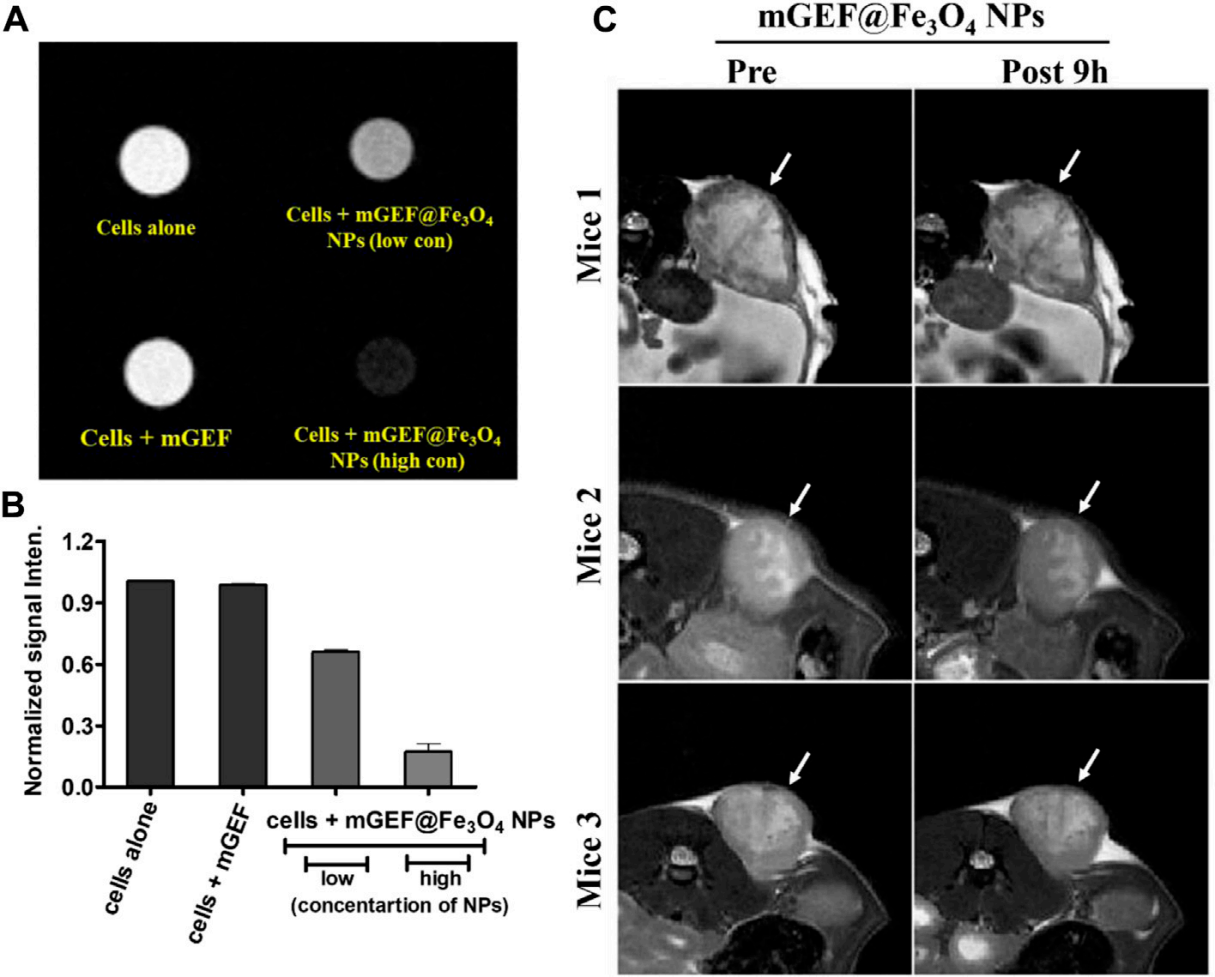
*In vitro* and *in vivo* molecular imaging of PC9 NSCLC on mGEF@Fe_3_O_4_ NPs. **(A)** *In vitro* MRI phantom images of PC9 cells treating with mGEF alone and mGEF@Fe_3_O_4_ NPs (low and high concentrations of *on mGEF@Fe*_*3*_ *O*_*4*_ *NPs* are respectively 0.196 μM and 0.39 μM in terms of Fe concentration). **(B)** Corresponding kinetic plot of contrast enhancement at different conditions. **(C)** *In vivo* T2 weighted negative contrast enhancement of tumor post-9 h injection of mGEF@Fe_3_O_4_ NPs (injection, dose of 30 mg of [Fe]/kg). All *in vivo* group experiments used three mice (*n* = 3); error bars show standard deviations of three repetitions of each experiment.

**FIGURE 4:**
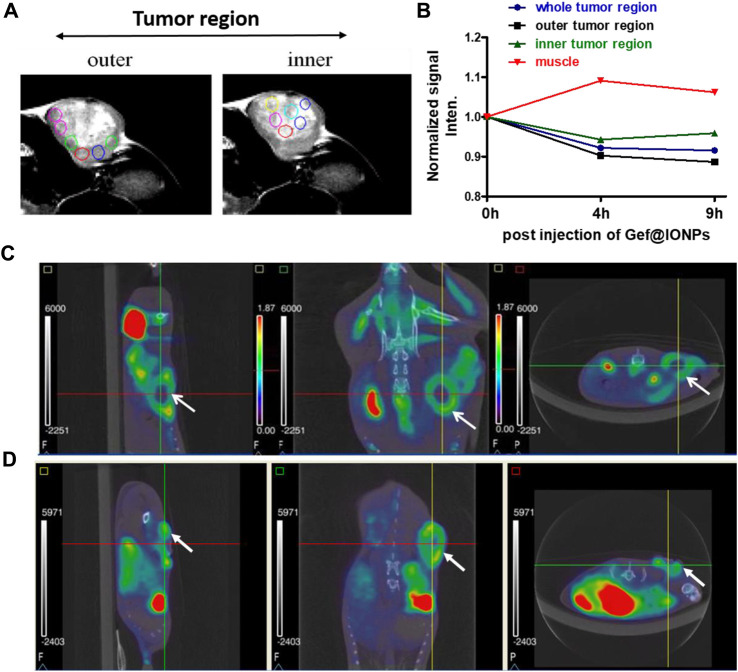
Monitoring the inner vs. outer tumor contrast. **(A)** MRI images of *in vivo* tumor indicating inner and outer tumor region. **(B)** Kinetic plot of T2 contrast intensities in muscle, inner and outer tumor region after post injection of mGEF@Fe_3_O_4_ NPs. **(C, D)** Respectively PET/CT imaging of FDG uptake and FLT uptake on PC9 xenograft model. Left to Right: sagittal, coronal and axial views, with arrow to indicate tumor. All *in vivo* group experiments used three mice (*n* = 3); error bars show standard deviations of three repetitions of each experiment.

Thereafter, mGEF@Fe_3_O_4_ NPs’ MR imaging capabilities were assessed both *in vitro* and *in vivo*. First, to assess the MR contrast effect of mGEF@Fe_3_O_4_ NPs within the cells, various concentrations of mGEF@Fe_3_O_4_ NPs were incubated with PC9 cells for a given time for the MR imaging experiments. As shown in Figure 3A, no significant changes were noticed in the case of cells incubated with mGEF and cells alone. In contrast, cells treated with mGEF@Fe_3_O_4_ NPs darken significantly than other control groups due to the T2 effect. Moreover, increasing concentrations of mGEF@Fe_3_O_4_ NPs dramatically changes the signal intensity of the T2-weight phantom images and increases the darkness. The difference in signal intensity reached 35% (at low conc.) and 87% (at high conc.) respective to the control group, as shown in Figure 3B. *In vitro* contrast ability studies demonstrated the successful internalization of mGEF@Fe_3_O_4_ NPs into cancer cells, indicating feasibility for T2-enhanced MR imaging applications. Furthermore, *in vivo* MR imaging was performed by establishing the xenograft PC9 tumor-bearing animal model by subcutaneously injecting 4 ×10^6^ PC9 cells (in 0.1 mL PBS) at the mouse’s left back (Supplementary Figure S9). Two weeks after tumor implantation, mGEF@Fe_3_O_4_ NPs (30 mg OF [Fe]/kg) were injected intravenously via the tail vein. Thereafter, *in vivo* MR imaging was performed at different time points (pre- and post 9 h-injection of mGEF@Fe_3_O_4_ NPs). Figure 3C shows the MR images of mice (*n* = 3) pre- and post-injection of mGEF@Fe_3_O_4_ NPs. 9 h post-injection, the signal intensity in the tumor was significantly lower than in the control group, which indicates that the accumulation of NPs at the tumor site corresponding to the successful delivery of chemo drug to the tumor increased locally. As a result, contrast intensity gradually decreased correspondingly to the T2 effect by increasing the post-injection time of mGEF@Fe_3_O_4_ NPs. The signal intensity at the tumor region was decreased to 15% after 4–9 h of post admisnstartion of NPs, and after 24 h it gradually dropped to 23% (Supplementary Figure S10). Even though *in vivo* imaging showed a significant accumulation of Fe_3_O_4_@gefitinib NPs at the tumor, the degree of enhancement is not as significant as expected, probably due to the significant differences of contrast between the inner and outer regions of the tumor 9 h post-injection of mGEF@Fe_3_O_4_ NPs. The negative enhancement of the inner region was not as obvious as the outer region, and the inner was brighter than the outer tumor in T_2_-weighted MR images. Thus we segmented the tumor into outer and inner regions and measured the contrast change at the different time points (Figures 4A, B). After 9 h of mGEF@Fe_3_O_4_ NPs injection, the signal intensity of the outer tumor region decreased by 12% whereas the inner tumor region only decreased by 4%. The inner region was proton-rich so the signal intensity was higher than around the tissue in the T_2_-weighted imaging. According to our hypothesis, the inner region had undergone necrosis. Cells swelled and even disintegrated after the intracellular contents were released, so the images showed hyperintensity in T2-weighted imaging. Consequently, it was difficult to deliver the mGEF@Fe_3_O_4_ NPs into the inner region where vasculature was lacking. As proof of concept, we analyzed and compared the histology of the outer and inner tumor regions. As shown in Supplementary Figures S11, S12, cells of the inner tumor (left) were shrunken with minimal amounts of iron, whereas the cells located in the outer region (right) retained their shape with intensive iron deposits. H&E staining analysis of the tumor region (Supplementary Figure S13) revealed distinct differences between the morphologies of the inner and outer regions, where the cells of the outer region were crowded and intact, while those of the inner region were arranged loosely and shrunken, indicating that the inner region cells underwent greater necrosis.

To further investigate he differences between outer and inner tumor regions, we also performed PET/CT scan imaging to obtain functional information such as staging, glucose metabolism and cell cycle physiology. We separately used the [^18^F] fluorodeoxyglucose (FDG) and [^18^F] fluoro-L-thymidine (FLT) as a tracer for PET scanning (Rasey et al., 2002; Ullrich et al., 2008). The tracer fluorine-18 [^18^F]) fluorodeoxyglucose (FDG) is a glucose analog that is taken up by glucose-using cells and phosphorylated by hexokinase, the mitochondrial form of which is greatly elevated in rapidly growing malignant tumors (Czernin et al., 2013; Kawada et al., 2016). The [^18^F]-fluoro-L-thymidine ([^18^F]FLT) is a specific marker to measure cellular proliferation *in vivo*. As an analog substrate of thymidine, [^18^F] FLT is phosphorylated by thymidine kinase 1 which is a cytosolic enzyme synthesized when proliferating cells enter the S-phase for DNA synthesis. First, we measured the maximum standard uptake value (SUV) of the tumor and the normal tissue using dynamic PET imaging (Supplementary Figure S14). The time-activity curve (TAC) shows the SUV of normal tissue decreases over time, while the tumor uptake remained consistent 0–120 min post-injection, and the contrast between the tumor and normal tissue is gradually enhanced. The obvious difference is found at 120 min after injection, indicating the suitable image acquisition time for distinguishing tumor from normal tissue is 60–120 min after tracer injection. Figures 4C, D show the[^18^F] FDG and [^18^F] FLT PET/CT scan results on PC9 NSCLC mice model. In [^18^F] FDG or [^18^F] FLT images, the tumor site presented a circular donut-shaped hot spot (tumor region indicated with an arrow). The γ-ray emission means *in vivo* biodistribution of the tracer, and the radioactivity of the inner region was so low even near the background, indicating that the tracer could not be transported into the inner side or the dead cell is disabled to take up the tracer. In conclusion, the inner tumor was necrosed and deficient in activity. Because of the tumor necrosis at the inner region, there was no blood vessel to deliver material including radioactive tracers or Fe_3_O_4_@gefitinib, sufficiently explaining that MR imaging could be enhanced only at the outer region of the tumor.

### 2.4 *In vivo* biodistribution of Fe_3_O_4_ NPs

Biocompatibility plays a vital role before prior to the implementation of biomedical health applications (Thangudu et al., 2022a). In the former section, we demonstrate *in vitro* that the self-synthesized Fe_3_O_4_ NPs are nontoxic and efficiently enhanced MRI tissue contrast. Although the biocompatibility of iron oxide nanoparticles is well-known, we are still concerned with potential excretion. Following intravenous administration of Fe_3_O_4_ NPs, we used the 7T MRI to observe the contrast in mice liver (*n* = 3) at different time points up to a week (Supplementary Figure S15). The Fe_3_O_4_ NPs gradually accumulated in the liver, hence the average signal intensity decreased over time, the liver contrast in MRI was retrieved to pre-administration, and we can speculate that NPs accumulated in the liver were further excreted over the course of the week. Furthermore, we evaluated the biodistribution of Fe, performing histological Perl’s blue staining assay after Fe_3_O_4_ NPs administration via iv injection. At different post-injection time points (1.5 h, 3, 6, and 9 days) the animals were sacrificed and livers were collected, embedded in paraffin, and sectioned for Perls’ blue staining (Supplementary Figure S16). This assay will be helpful to detect iron selectively, with iron appearing blue and the cytoplasm pink. Fe deposition in the liver region was substantially increased post-1h NPs injection. After prolonged post-injection time (from 3–6 days), Fe in the liver region gradually decreased and, on the 9-day post-injection, no noticeable Fe content was observed in the liver region. These results revealed the Fe_3_O_4_ NPs are biocompatible and excreted after treatment without creating adverse effects on the major organs.

## 3 Conclusion

In conclusion, we have successfully integrated mGEF@Fe_3_O_4_ NPs by chemically conjugating the synthesized mGEF chemo drug to Fe_3_O_4_ NPs. The present direct chemical conjugation of the drug with NPs significantly boosted the loading efficiency resulting in more efficient drug internalization by PC9 lung cancer cells followed by improved chemotherapeutic efficacy, estimated IC 50 value on mGEF@Fe_3_O_4_ NP is 2.0 μM. Furthermore, MRI and PET/CT imaging analysis was performed and reveals that more NPs were accumulated in the outer region of the tumor than in the inner region due to the necrosis at the inner tumor region. *In vivo* biodistribution analysis showed that NPs were successfully excreted after treatment and had no adverse effects on the body’s primary organs. Overall, combining chemically conjugated drug delivery on NPs with an image-guided technique can solve the problems associated with drug leakage as well as understanding the therapeutic prognosis of tumor to further accelerate the chemotherapy efficacy in upcoming cancer treatment applications.

## 4 Experimental section

### 4.1 Synthesis of -NH_2_—*Fe*_*3*_ *O*_*4*_ *NPs*

The preparation of NH_2_—*Fe*_*3*_ *O*_*4*_ *NPs* was carried out as previously described (Su et al., 2016). 1 M ferric chloride hexahydrate (FeCl_3_·6H_2_O) and 2.0 M ferrous chloride tetrahydrate (FeCl_2_·4H_2_O) were prepared by separatly dissolving in 2 M HCL solution. After that, 4 mL of FeCl_3_·6H_2_O was mixed with 1 mL of FeCl_2_·4H_2_O solution and then 1 mL of a organic acid solution was added under constant stirring. Subsequently, the pH of the solution was carefully adjusted to 13 by adding 5 M NaOH followed by the proper amount of adherent to achieve -NH_2_—*Fe*_*3*_ *O*_*4*_ *NPs*.

### 4.2 Modified GEF conjugated Fe_3_O_4_ NPs (mGEF@Fe_3_O_4_ NPs)

In a typical preparation, the modified drug (40 μM) was dissolved in 1 mL DMSO solution, followed by the addition of Fe3O4 nanoparticles (40 nM) and EDC with overnight stirring at room temperature (Su et al., 2016). The resulting products were subjected to five centrifugation/re-suspension with a DMSO solution followed by water to remove the excess or unbonded drug molecules and EDC. Finally, Fe_3_O_4_-drug NPs were resuspended in 0.2 mL DMSO solution and stored. For further biological tasks *in vitro* and *in vivo*, the surface of Fe_3_O_4_@gefitinib was modified with PEG (MW: 4000) to improve the solubility of gefitinib in water.

### 4.3 *In vitro* toxicity evaluation

The toxicity of iron oxide nanoparticles was assessed by MTT assay using the same protocol as that used for drug efficiency evaluation. PC9 cells were cultured in a 96-well at an initial density of 4 ×10^3^ cells/well. After 24 h, different concentrations of the Fe_3_O_4_ nanoparticles were added to the cells with final concentrations ranging from 0.257 to 2570 μM. After 24 h incubation, the culture medium was removed and 3-[4,5-dimethylthiazol-2-yl] −2,5-diphenyl tetrazolium bromide (MTT) reagent (0.5 mg/mL, Sigma) was added, followed by another 1.5-h incubation to allow the formation of formazan dye. Next, the culture medium was removed, and the DMSO (100 μL/well) was added across the plate. Cell viability was quantified using an ELISA plate reader to acquire 570 nm optical absorbance. PC9 (human non-small-cell lung adenocarcinoma) cell lines present in this study were obtained from American Type culture Collection (ATCC).

### 4.4 Cell viability studies

We used both the GEF drug-sensitive (PC9) and the resistant (A549, CL1-0, CL1-0 transfect VEGF isoform 189 and CL1-5, all wild type) cell lines to assess drug efficiency. The cell lines present in this study were obtained from American Type culture Collection (ATCC).

Briefly, different cancer cells were cultured in a 96-well at an initial density of 4 ×10^3^ cells/well. To measure drug targeting performance, different concentrations of mGEF in 100 μL of DMSO (0.1 nM–100 μM) were added to the culture wells. After 24 h incubation, the culture medium was removed and MTT reagent (0.5 mg/mL, Sigma) was added, followed by another 1.5-h incubation to allow the formation of formazan dye. Next, the culture medium was removed and the DMSO (100 μL/well) was added across the plate. The quantification determining cell viability was using an ELISA plate reader to acquire 570 nm optical absorbance.

To measure the mGEF@Fe_3_O_4_ NPs drug activity, PC9 and A549 cancer cells were seeded in a 96-well plate (4 ×10^3^ cells/well). After 24 h, different concentrations of the mGEF@Fe_3_O_4_ NPs were added to the cells with final concentrations ranging from 0.0 to 100 μM, and activity levels were measured using an ELISA plate reader to acquire 570 nm optical absorbance.

### 4.5 *In vitro* Perl’s blue staining

The cells were seeded on the 4-well chamber slides (Millipore, Millicell EZ slide) in a density of 1.55 × 105/0.1 mL/well. After the cells were attached at the bottom of the dish (about 24 h), we added the prepared nanoparticles-drug into one well of the chamber slide. After at diffrent time of incubations, perls’ blue stain was used to observe the nanoparticles endocytosed into the intracellular domain. Before staining, PBS washed the cell twice to wash out the nanoparticle-drug at the extra-cellular domain. The cells were fixed by 4% formalin for 20 min and PBS washed 3–5 times. The protocols of Perls’ blue stain are as follows (Su et al., 2016).

The sections were incubated in a solution of 4% potassium ferrocyanide solution and 4% HCl (1:1 mixed) for 10 min. After PBS washing, the cytoplasm was stained in pararosaniline solution (1 mL 1% hydrochloric acid solution diluted in 50 mL ddH2O) for 5 min, then rinsed in PBS to wash out the remaining solution. Next, we removed the cover chamber to access the microscope slide, and sections were dehydrated in 50%, 70%, 90%, and 100% EtOH and rinsed in xylene twice.

### 4.6 Annex V and PI staining

After 96 h of drug treatment, cells were washed twice with 4°C PBS. We then prepared the mixing solution (including Annexin V binding buffer (100 μL/well) and fluorochrome-conjugated Annexin V (5 μL/well), and PI solution (10 μL/well)) and added into each well. After 15 min, the fluorescence microscopy results depicted the Annexin V-FITC in green and the PI in red.

### 4.7 *In vitro* MR imaging

The PC9 cells were cultured in 10 cm-diameter dishes with an initial density of 5×10^6^ cells/well. After 1 day, the Fe3O4@gefitinib nanoparticles with 150 μM and 300 μM drug concentrations were added to the cells. We also treated the modified drug and medium (as control) in the other two dishes, so there is a total of four groups all treated for 24 h. The cells were washed twice with phosphate-buffered saline (PBS) and acquired the cells by 0.25% trypsinization. The cells were placed in a micro-tube and replenished with PBS. Just before MRI scanning, we centrifuged the cells down to the bottom of the tube and put four tubes in a holder immersed in mineral oil as previously described. In image acquisition, we selected the tubes that were full of cells at the bottom. The images were performed with a T2-weighted, T1-weighted sequence.

### 4.8 *In vivo* animal model

The procedures for all animals were carried out in accordance with the guidelines of the National Research Council (United States) and the Animal Welfare Regulations of the Council of Agriculture, Executive Yuan (R.O.C.). All experimental protocols involving live animals were reviewed and approved by the Chang Gung Memorial Hospital Institutional Animal Care and Use Committee (approval number: IACUC No. 2021031802). The tumor animal model was generated by subcutaneously injecting 4 ×10^6^ PC9 cells (in 0.1 mL PBS) at the mice left back. Two weeks later, the mice were subjected to MR imaging studies.

### 4.9 *In vivo* MR imaging

We used a 7.0 T MRI scanner to observe the *in vivo* image enhancement and performed experiments by following the previously reported protocol (Su et al., 2016). The Balb/C mice (male, 25–30 g) were anesthetized with 5% isoflurane, and a 30G needle connected to a syringe with a 50 cm polyethylene tube (PE8) was inserted in the tail vein. After acquiring the pre-imaging, we injected the prepared iron oxide nanoparticles dispersed in normal saline with 10 mg [Fe]/kg dosage and additionally injected 0.5 mL saline to wash the nanoparticles left in the polyethylene tube into the vein. The images were taken immediately, and 0.5 h followed up to 1.5 h. The signal intensity of the tissue was determined using standard region-of-interest measurements with homemade image quantification software. The image-acquiring pulse sequence is TurboRARE T2 pulse sequence (TR: 4000 ms, TE: 33 ms, FA: 180 deg, Matrix size: 256 × 256 × 17, FOV: 4 × 4 cm, NEX: 3, Thickness: 1 mm).

### 4.10 PET/CT imaging

The mice were briefly anesthetized using isoflurane gas, after which 350–390 μCi of ^[18F]^ FLT or ^[18F]^ FDG tracer was injected through the tail vein. After a 60-min conscious uptake period, we performed CT scanning and a 60-min PET acquisition, with images covering the area from the diaphragm to the coccyx (2D mode, one-bed positions). Emission data were corrected for random coincidences and scatter coincidences dead time, then reconstructed by OSEM. The matrix size was 175 × 175 pixels with a pixel size of 0.3875 × 0.3875 mm.

### 4.11 Perl’s blue staining

The paraformaldehyde-fixed paraffin-embedded organs were sectioned and mounted on glass slides, followed by drying in an oven for 1 h at 65 C. Subsequently, an iron working solution was prepared by mixing equal volumes of hydrochloric acid solution and potassium ferrocyanide solution (HT20; SigmaAldrich). Later, all the organ-coated glass slides were treated with a working solution for 10 min followed by water wash and treated with a working pararosaniline solution for 2 min. All the slices were then washed with deionized water for 2 min and then rapidly dehydrated using alcohol and xylene. Finally, images were acquired using a microscope (Olympus BX51; Yuan Li Instrument Co., Taipei, Taiwan).

### 4.12 H&E staining

After the scanning, the mouse was sacrificed and the tumor was excised for further paraffin embedding, followed by H&E staining.

### 4.13 Statistical analysis

All data will be presented as the mean ± standard error and compared between groups using the Student’s t-test or one-way ANOVA using GraphPad Prism 5 Software (GraphPad Software, Inc., San Diego, CA). A *p*-value of <0.05 will be considered statistically significant. *: *p* < 0.05; **: *p* < 0.01; ***: *p* < 0.001.

## Data Availability

The datasets presented in this study can be found in online repositories. The names of the repository/repositories and accession number(s) can be found in the article/Supplementary Material.
