# Supplementary material for: Modified gefitinib conjugated Fe3O4 NPs for improved delivery of chemo drugs following an image-guided mechanistic study of inner vs. outer tumor uptake for the treatment of non-small cell lung cancer
Source: Front Bioeng Biotechnol. 2023 Oct 9;11:1272492. doi: 10.3389/fbioe.2023.1272492 (PMC10591449; doi:10.3389/fbioe.2023.1272492)
Supplement: Supplementary file 1 [file Datasheet1.docx]

Supporting information

Modified Gefitinib conjugated Fe_3_O_4_ NPs for Improved Delivery of Chemo Drugs Following an Image-guided Mechanistic Study of Inner vs Outer Tumor Uptake for the Treatment of Non-Small Cell Lung Cancer

**Experimental section:**

**Synthesis process of Therapeutic drug (modified Gefitinib):** Synthesis of Gefitinib drug was achieved by using following steps. The 4-anilinoquinazoline **4** possessing the aliphatic acid chain at C-7 have been synthesized from the corresponding 7-benzyloxy-4-cholroquinazoline **1** in high yields. First, Coupling of **1** with 4-chloro-2-fluoroaniline under acid catalysis followed by deprotection of the C-7 benzyl moiety using TFA according to the method of Hennequin’s et al. [27] led to the corresponding unprotected C-7 hydroxy-4-anilinoquinazoline **2**. Subsequently, Direct alkylation of **2** with ethyl 6-bromohexanoate in the presence of K2CO3 in DMF to achieve **3** and followed by hydrolysis afforded the target compound **4**.

**Step-1:**

**N-7-Benzyloxy-(4-chloro-2-fluorophenyl)-6-methoxy-4-quinazolinylamine hydrochloride (1)**

A mixture of 7-benzyloxy-4-chloro-6-methoxyquinazoline hydrochloride (337 mg, 1.0 mmol) and 4-chloro-2-fluoroaniline (175 mg, 1.2 mmol) in 2-propanol (30 mL) was refluxed overnight. After cooling, the precipitate was collected by filtration, washed with 2-propanol and dried under vacuum to give pure 1. Yield 73%; white solid, mp 203–205 oC (lit.1 mp 239–242 oC). 1H NMR (DMSO-d6) δ 3.98 (3H, s), 5.33 (2H, s), 7.40 (4H, m), 7.50 (2H, d, J = 7.6 Hz), 7.54 (1H, d, J = 9.0 Hz), 7.67 (1H, m), 7.97 (1H, dd, J = 6.7, 2.5 Hz), 8.12 (1H, s), 8.81 (1H, s), 11.14 (1H, br s); 13C NMR (DMSO-d6) δ 56.8, 70.5, 102.3, 104.5, 107.5, 116.0 (d, J = 21.8 Hz), 118.7 (d, J = 18.3 Hz), 124.4, 125.7, 127.3, 127.7, 128.0, 134.3, 135.1, 136.7, 148.6, 150.3, 154.5 (d, J = 244.7 Hz), 155.0, 157.6; IR (KBr) 1631 cm-1.





**Step-2:**

N-(4-Chloro-2-fluorophenyl)-7-hydroxy-6-methoxy-4-quinazolinylamine (2).

A solution of 4-quinazolinylamine hydrochloride **1** (982 mg, 2.2 mmol) in TFA (10 mL) was refluxed for 1 h. After cooling, the mixture was poured onto ice. The precipitate was collected by filtration, dissolved in methanol and basified with aqueous ammonia. After concentration by evaporation, the solid product was collected by filtration, washed with H_2_O then ether and dried under vacuum to give **2** Yield 95%; white solid, mp 141–142 ^o^C (lit.^1^ mp 141–143 ^o^C). ^1^H NMR (DMSO-*d*_6_) *δ* 3.95 (4H, s), 7.08 (1H, s), 7.43 (1H, t, *J* = 9.1 Hz), 7.74 (1H, m), 7.79 (1H, s), 8.41 (1H, dd, *J* = 6.8, 2.6 Hz), 8.45 (1H, s), 9.71 (1H, br s); ^13^C NMR (DMSO-*d*_6_) *δ* 56.5, 102.9, 107.8, 108.0, 116.9 (d, *J* = 21.6 Hz), 119.2 (d, *J* = 18.3 Hz), 123.4 (d, *J* = 6.9 Hz), 124.6, 136.3, 143.5, 149.5, 151.4, 154.0 (d, *J* = 242.4 Hz), 154.3, 156.9; IR (KBr) 3243, 3124, 1638 cm^-1^.





**Step-3:**

Ethyl 6-[4-(4-Chloro-2-fluorophenylamino)-6-methoxyquinazolin-7-yloxy]-hexanoate (3).

A mixture of 7-hydroxy-6-methoxy-4-quinazolinylamine 2 (959 mg, 3.0 mmol), ethyl 6-bromohexanoate (803 mg, 3.6mmol), and K_2_CO_3_ (1.04 g, 7.5mmol) in dry DMF (18 mL) was stirred at room temperature overnight. The mixture was added onto H2O (60mL). The precipitate was collected by filtration, washed with H2O then ether and dried under vacuum to give 3. Yield 77%; white solid, mp 164–165 oC. 1H NMR (CDCl3) δ 1.24 (3H, t, J = 7.1 Hz), 1.49 (2H, m), 1.66 (2H, m), 1.84 (2H, m), 2.32 (2H, t, J = 7.4 Hz), 3.81 (3H, s), 4.01 (2H, t, J = 6.6 Hz), 4.12 (2H, q, J = 7.1 Hz), 7.06 (1H, t, J = 8.8 Hz), 7.15 (1H, s), 7.33 (1H, s), 7.48 (1H, m), 7.79 (1H, m), 8.38 (1H, br s), 8.64 (1H, s); 13C NMR (CDCl3) δ 14.1, 24.5, 25.4, 28.3, 34.1, 56.0, 60.2, 68.7, 100.1, 107.9, 108.9, 116.3 (d, J = 22.0 Hz), 120.7 (d, J = 18.5 Hz), 121.9 (d, J = 6.6 Hz), 124.3, 135.4, 147.2, 149.7, 153.2, 154.3, 154.6 (d, J = 244.9 Hz), 156.4, 173.7; IR (KBr) 3306, 3121, 2945, 1728, 1624 cm^-1^.

**Step-4:**

6-[4-(4-Chloro-2-fluorophenylamino)-6-methoxyquinazolin-7-yloxy]-hexanoic acid (4).

A solution of 0.12N KOH (10 mL) was added to a solution of ester **3** (231 mg, 0.5 mmol) in EtOH (20 mL) and the reaction mixture was refluxed for 3 h. After cooling, the solution was evaporated, and the reside was dissolved in H_2_O, and acidified with 10% HCl. The precipitate was filtered, washed with CHCl_3_ and dried under vacuum to give **4**. Yield 98%; white solid, mp 248 ^o^C (decomp.). ^1^H NMR (DMSO-*d*_6_) *δ* 1.45 (2H, m), 1.59 (2H, m), 1.79 (2H, m), 2.24 (2H, t, *J* = 7.3 Hz), 3.95 (3H, s), 4.12 (2H, t, *J* = 6.4 Hz), 7.17 (1H, s), 7.43 (1H, t, *J* = 9.1 Hz), 7.78 (2H, m), 8.11 (1H, dd, *J* = 6.8, 2.5 Hz), 8.49 (1H, s), 9.58 (1H, s), 12.0 (1H, br s); ^13^C NMR (DMSO-*d*_6_) *δ* 24.3, 25.2, 28.2, 33.7, 56.3, 68.4, 101.8, 107.6, 108.6, 116.6 (d, *J* = 21.2 Hz), 118.8 (d, *J* = 18.2 Hz), 122.4 (d, *J* = 6.6 Hz), 123.5, 136.8, 146.7, 149.2, 152.5, 153.2 (d, *J* = 241.0 Hz), 153.8, 156.1, 174.5; IR (KBr) 3472, 1722, 1639 cm^-1^.





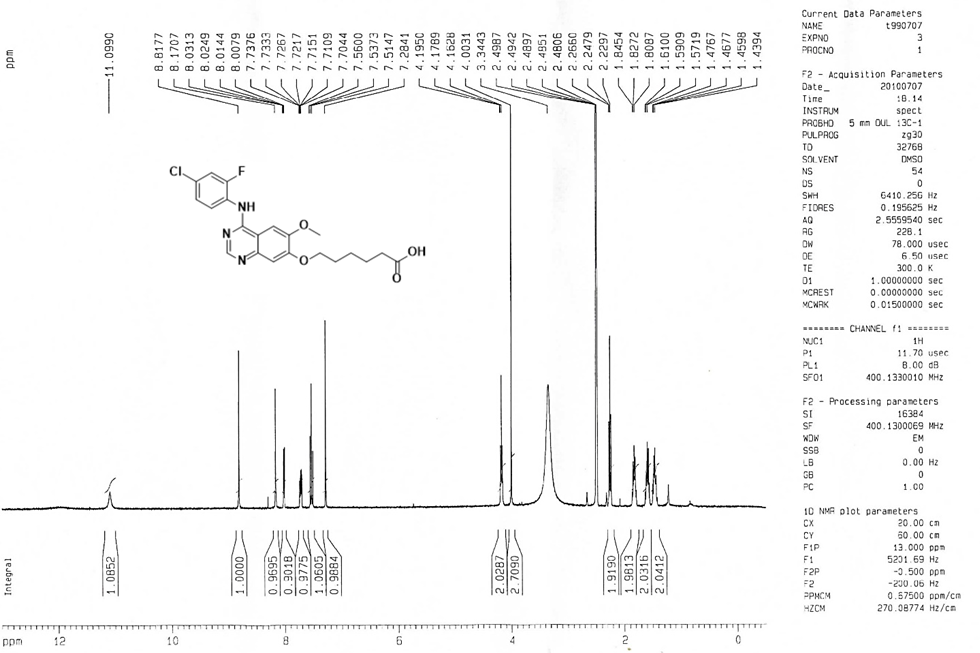


**Figure S1**. ^1^H NMR of modified gefitinib


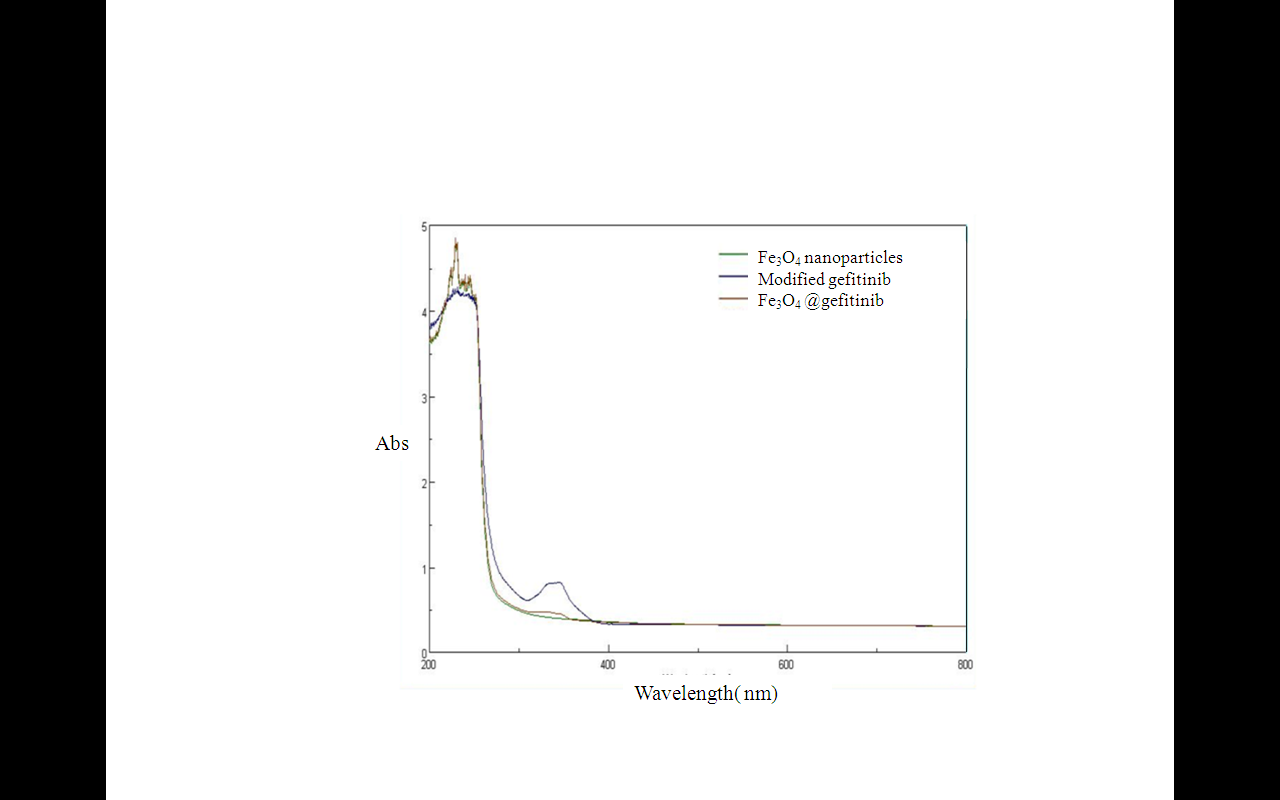


**Figure S2**. UV-Vis absorbance spectra of Fe_3_O_4_ nanoparticles (green), modified gefitinib (blue) and Fe_3_O_4_@gefitinib (dark-red).


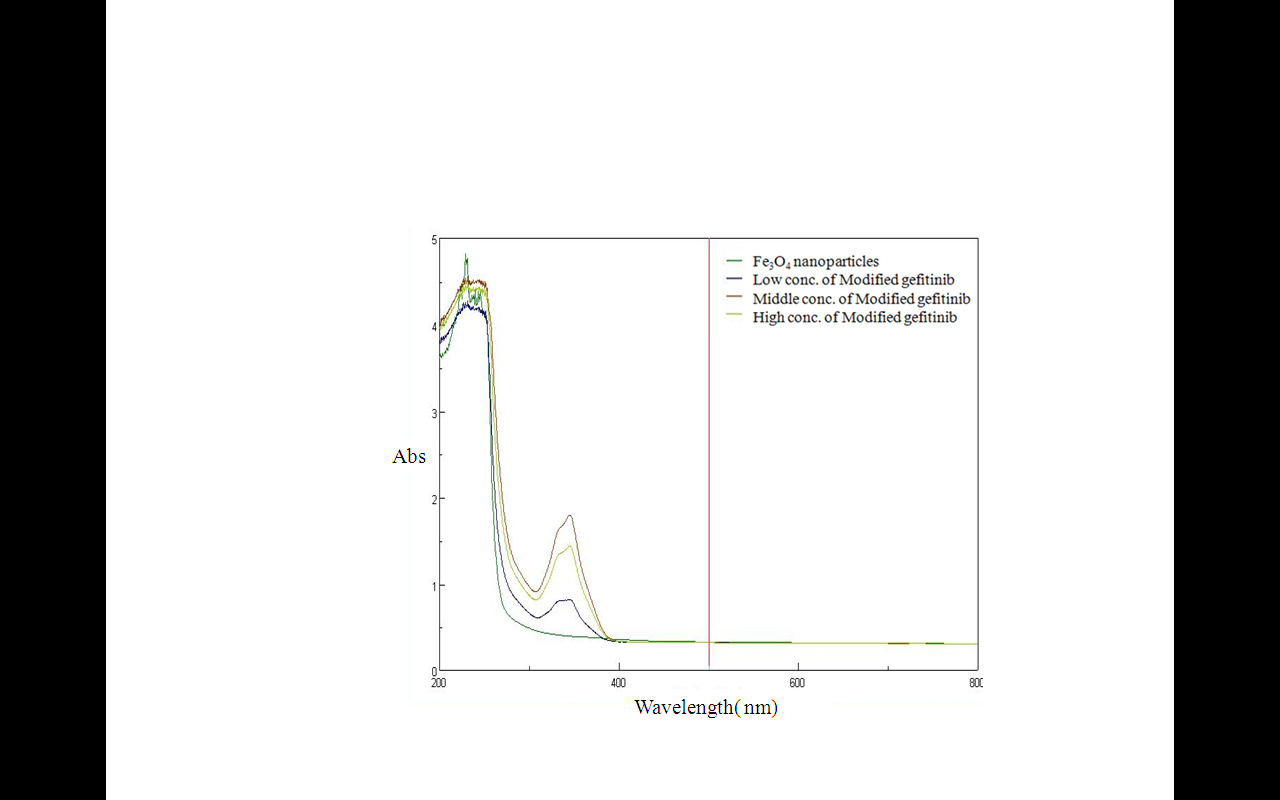


**Figure S3**. UV-Vis absorbance spectrum of Fe_3_O_4_ nanoparticles and modified gefitinib at different concentrations of mGEF drug.

**Figure S4**. Cytotoxicity evaluation of DMSO to PC9 cells.


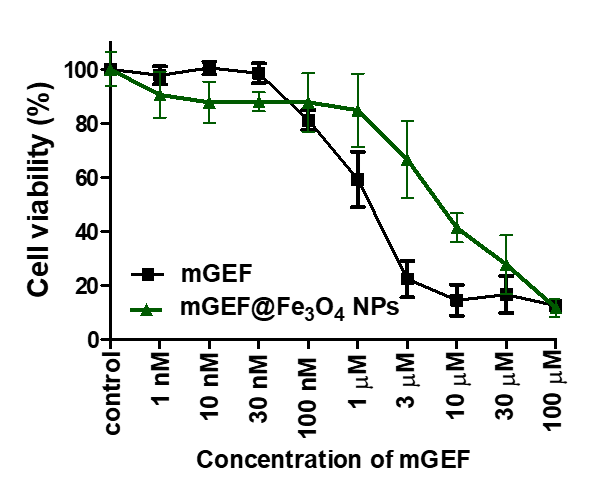


**Figure S5**. *In vitro* drug efficiency of mGEF and mGEF@Fe_3_O_4_ NPs.


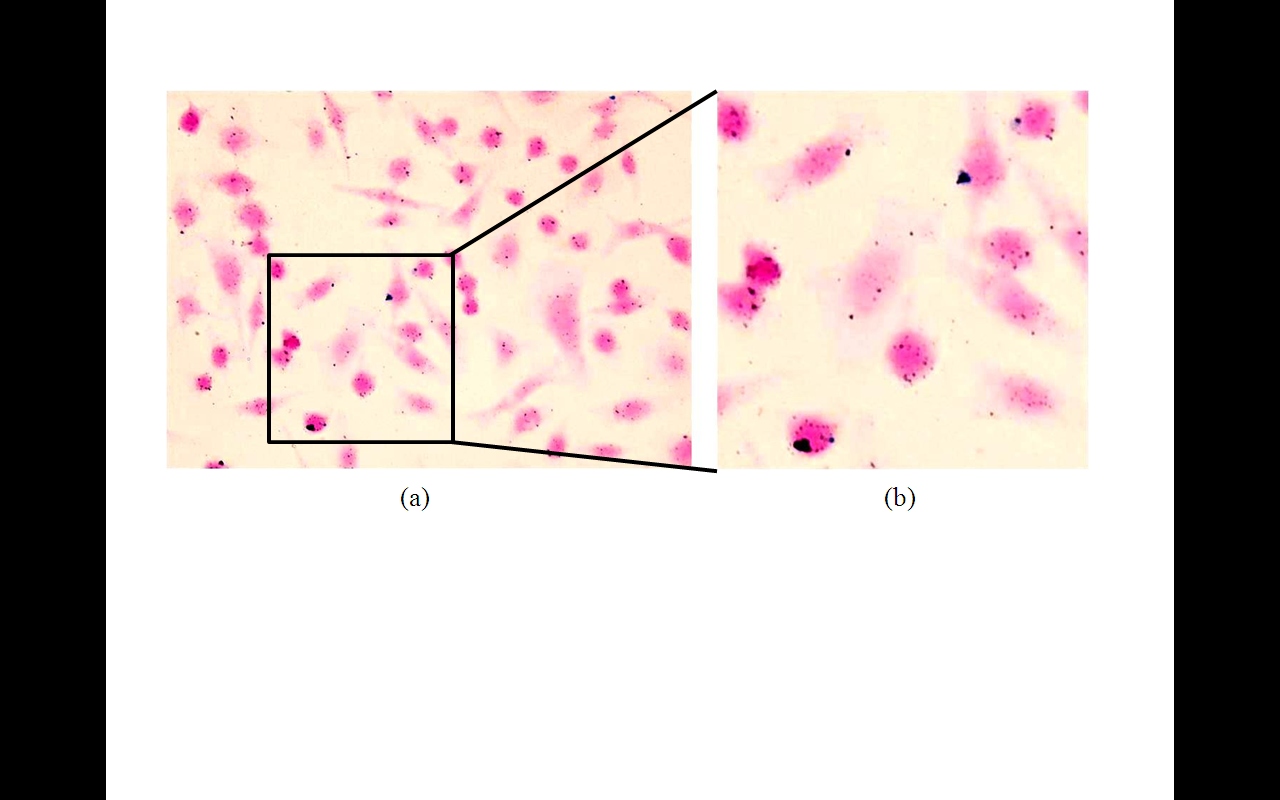


**Figure S6**. Magnified image of perls’s blue staining assay to confirm the endocytosis of mGEF@Fe_3_O_4_ NPs.


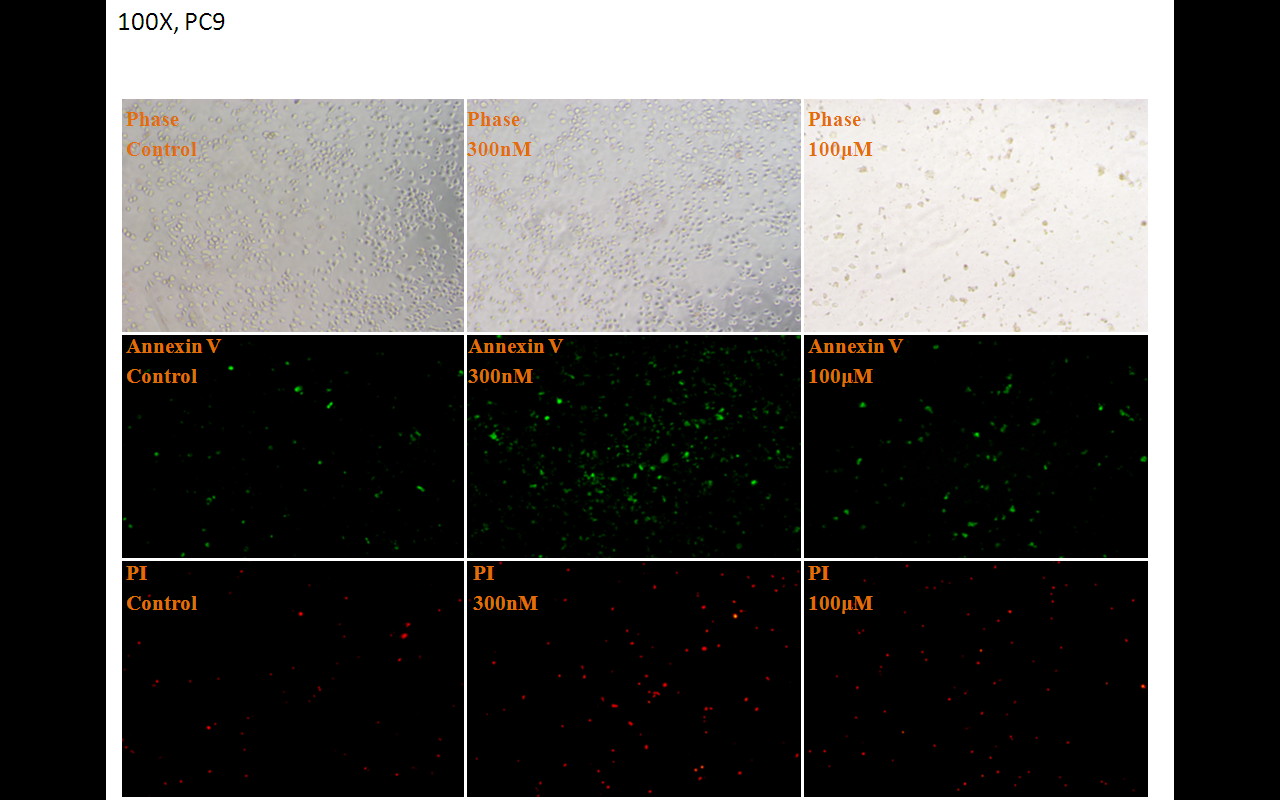


**Figure S7**. The 100X phase images(up), Annexin V-FITC(middle) and PI(bottom) staining of PC9 cells. From left to right, the control group(non-treating), 300 nM and 100 μM mGEF@Fe_3_O_4_ NPs treating respectively. The images of each row were photographed at the same FOV.


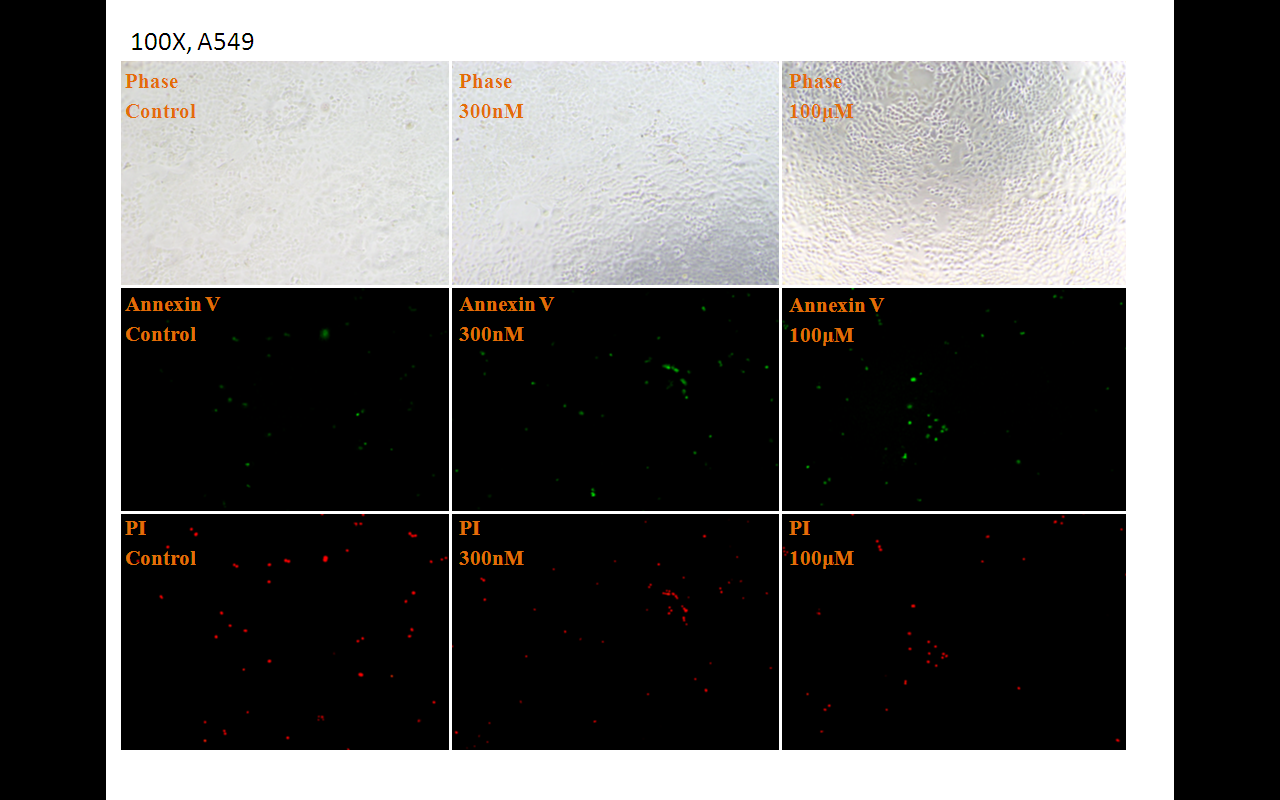


**Figure S8**. The 100X phase images(up), Annexin V-FITC(middle) and PI(bottom) staining of A549 cells. From left to right, the control group(non-treating), 300 nM and 100 μM mGEF@Fe_3_O_4_ NPs treating respectively. The images of each row were photographed at the same FOV.


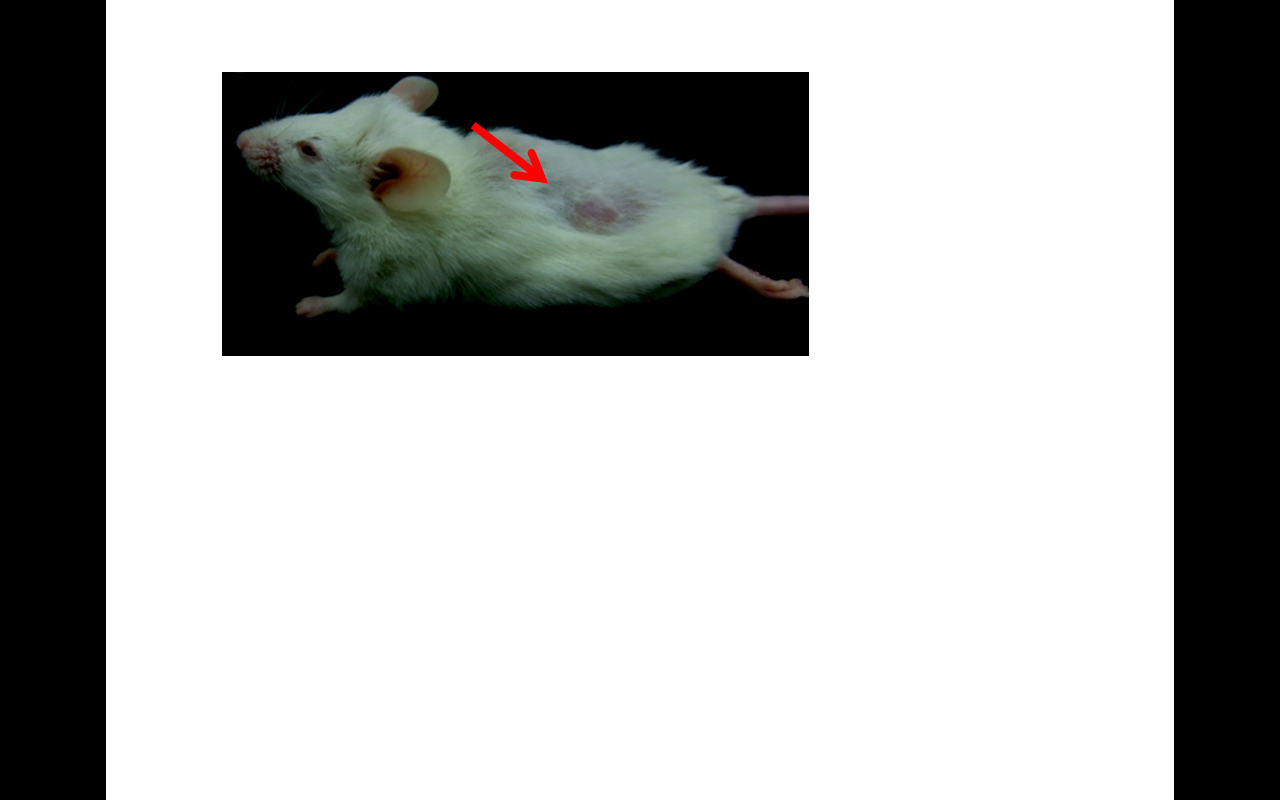


**Figure S9**. The xenograft PC9 tumor-bearing animal model.


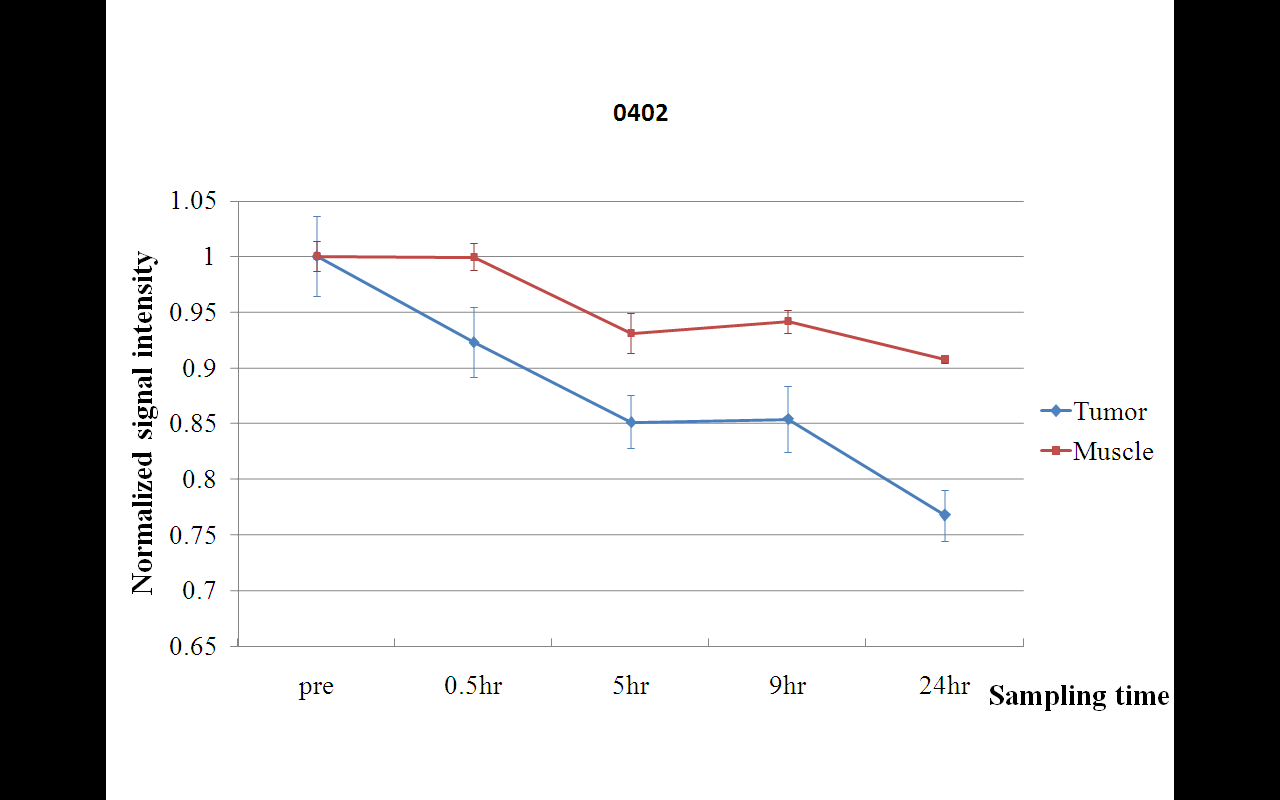


**Figure S10**. The tumor and muscle signal intensity change after the mGEF@Fe_3_O_4_ NPs injection.


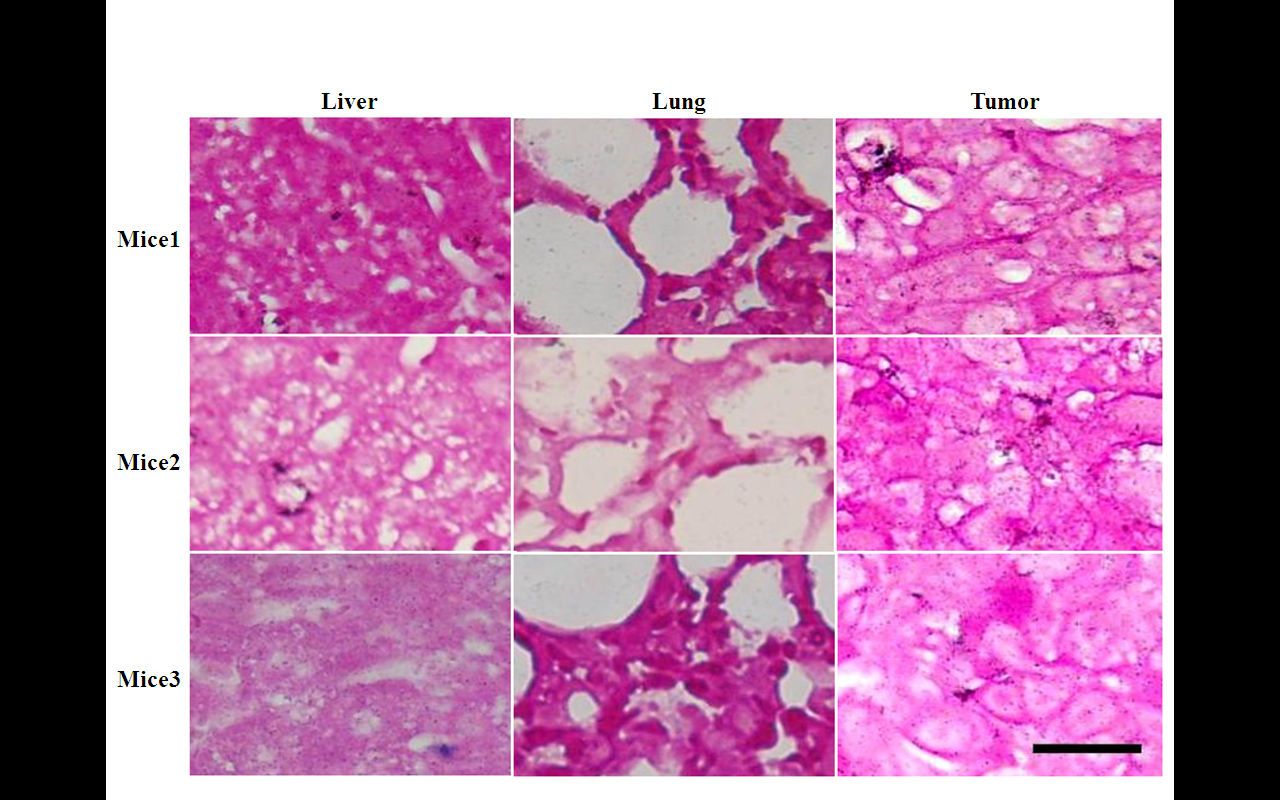


**Figure S11**. The Perls’ blue staining of mice organs after post-9 h injection of mGEF@Fe_3_O_4_ NPs, the mice were sacrificed and organs were collected for paraffin embedding. (scale bar was 25 μm).


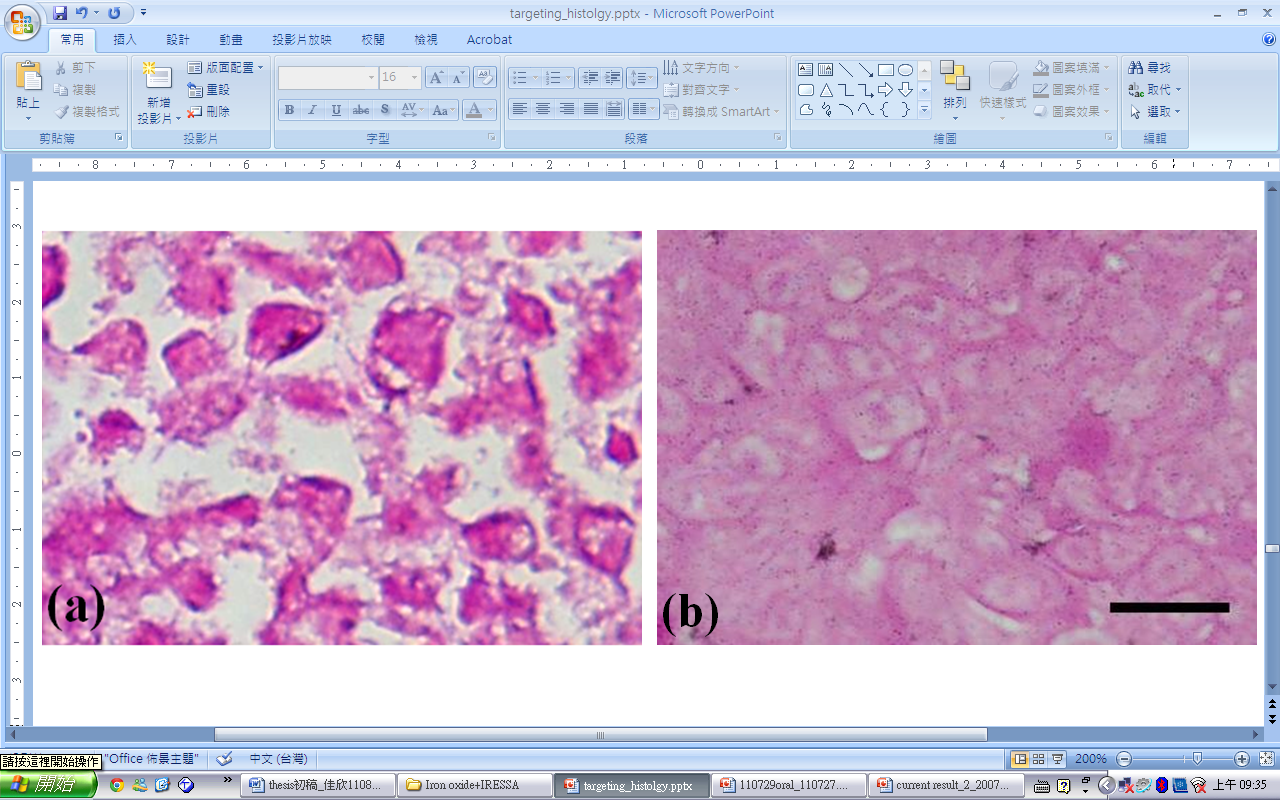


**Figure S12**. The Perl’s blue staining of tumor after post-9 h injection of mGEF@Fe_3_O_4_ NPs. (a) inner region. (b) outer region. (scale bar was 25 μm).


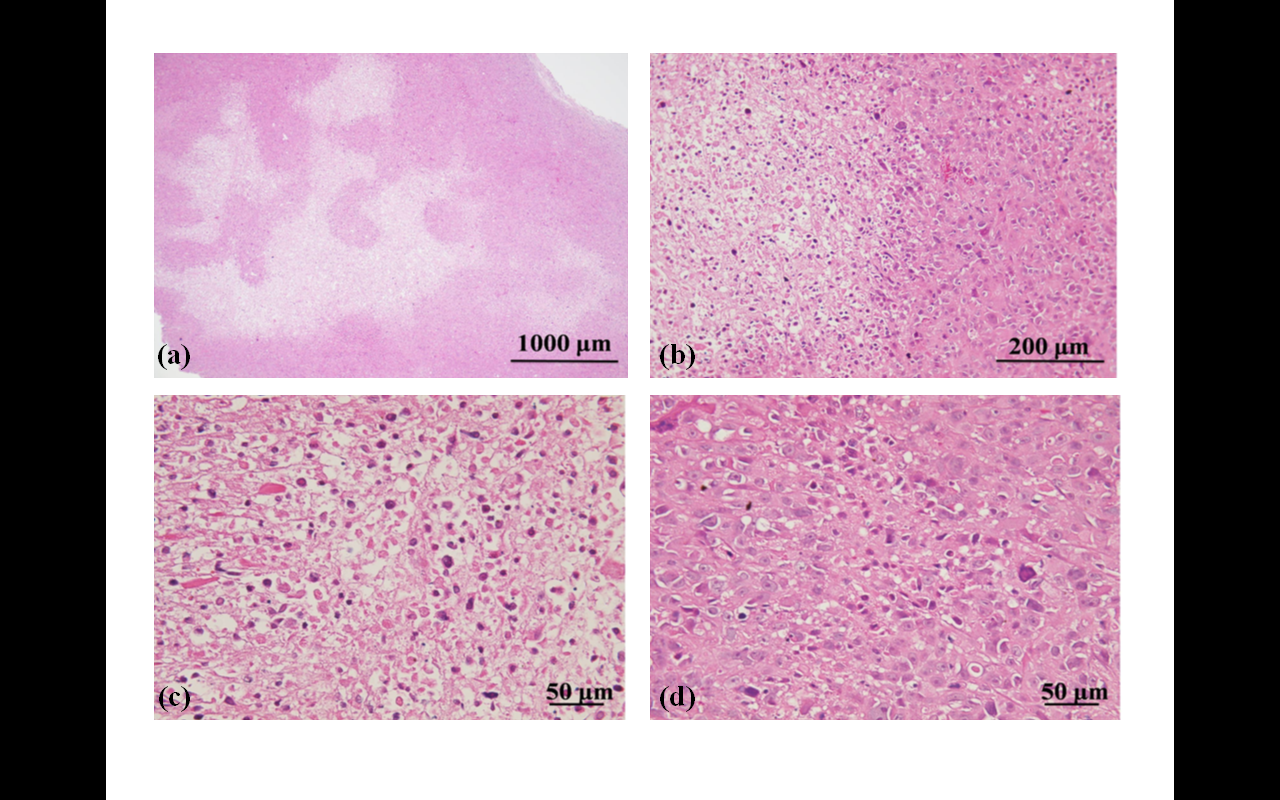


**Figure S13**. The H&E staining of tumor after post-9 h injection of mGEF@Fe_3_O_4_ NPs. (a) 40X magnification. (b) 100X magnification. The border of inner and outer region of tumor. Left: the inner region. Right: the outer region. (c) 400X magnification. The inner region of tumor. (d) 400X magnification. The outer region of tumor.


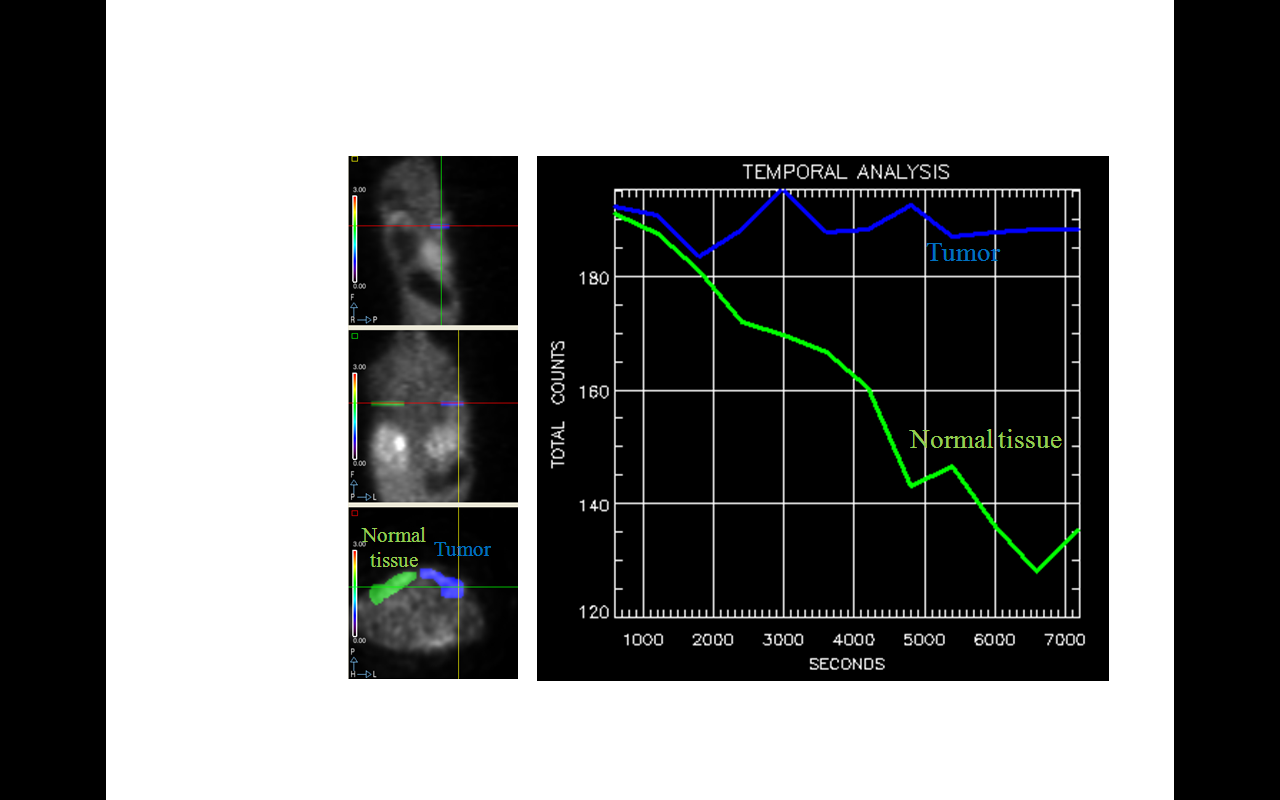


**Figure S14**. Time-activity curves for [^18^F]FLT uptake of tumor and normal tissue.

**
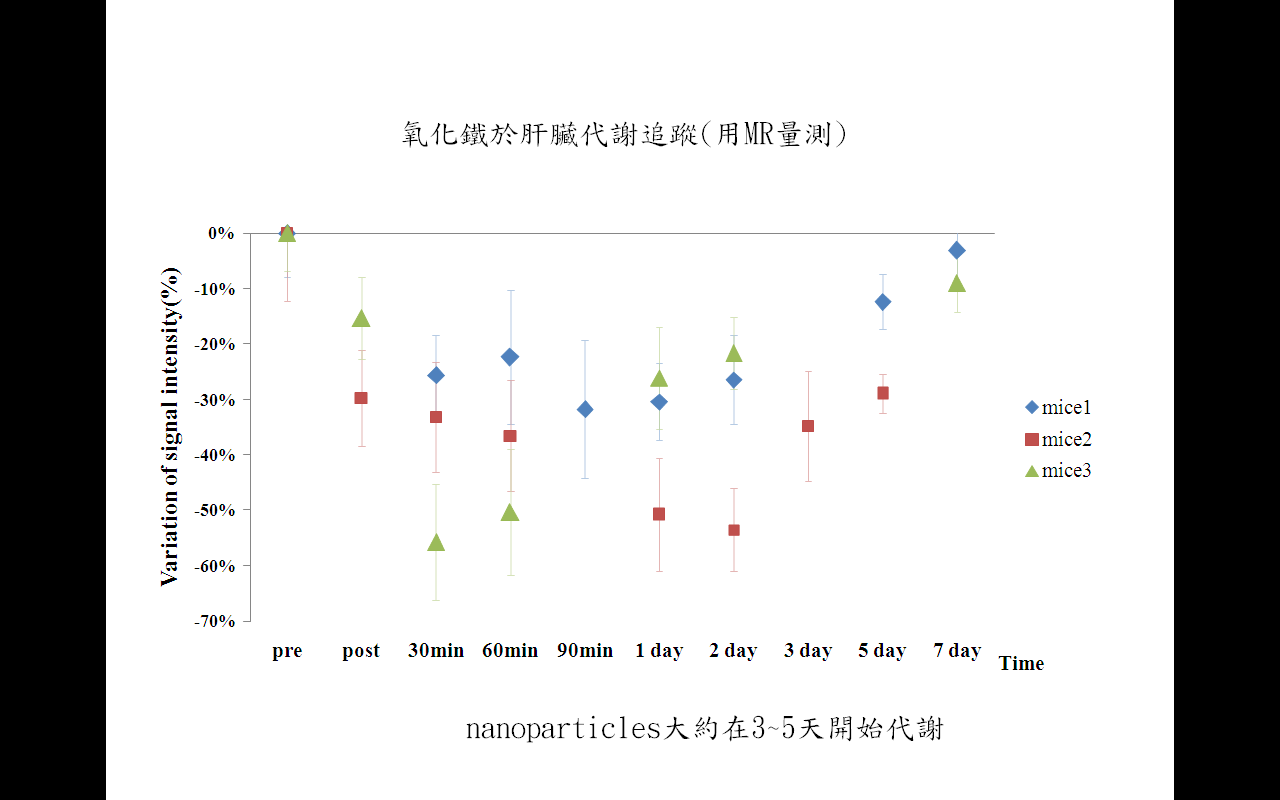
**

**Figure S15**. Time course of the Fe_3_O_4_ NPs excretion from the liver at different post injection points.


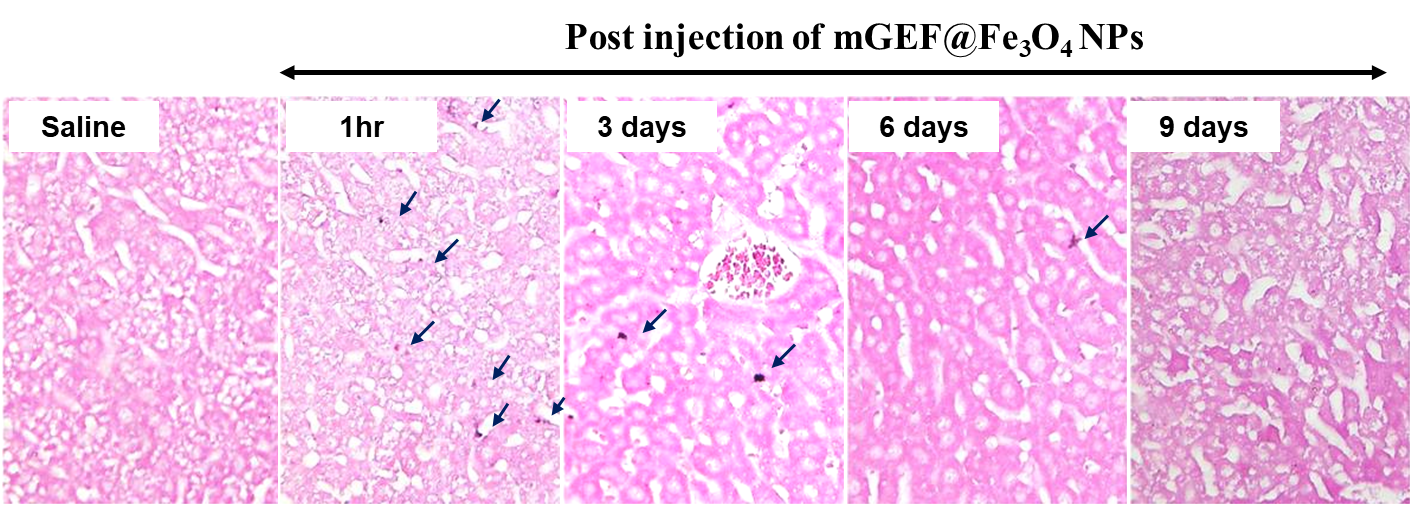


**Figure S16**. The Perls’ blue staining of mice liver at different post injection time points of Fe_3_O_4_ NPs.
